# A National Representative, Cross-Sectional Study by the Hellenic Academy of NeuroImmunology (HEL.A.NI.) on COVID-19 and Multiple Sclerosis: Overall Impact and Willingness Toward Vaccination

**DOI:** 10.3389/fneur.2021.757038

**Published:** 2021-11-25

**Authors:** Marina Boziki, Charis Styliadis, Christos Bakirtzis, Eleni Grigoriadou, Aggeliki-Styliani Sintila, Ioannis Nikolaidis, Aliki Vrienniou, Lotte Geys, Sygkliti-Henrietta Pelidou, Lesley Probert, Georgios Papazisis, Panagiotis Bamidis, Nikolaos Grigoriadis

**Affiliations:** ^1^2nd Neurological University Department, Aristotle University of Thessaloniki, American Hellenic Educational and Progressive Association (AHEPA) General Hospital, Thessaloniki, Greece; ^2^Hellenic Academy of NeuroImmunology (HEL.A.NI.), Thessaloniki, Greece; ^3^Laboratory of Medical Physics and Digital Innovation, School of Medicine, Aristotle University of Thessaloniki, Thessaloniki, Greece; ^4^Hellenic Federation of Persons With Multiple Sclerosis, Athens, Greece; ^5^University MS Center (UMSC), Campus Diepenbeek, Diepenbeek, Belgium; ^6^University of Hasselt, Biomedical Research Institute (BIOMED), Diepenbeek, Belgium; ^7^University of Hasselt, Data Science Institute (DSI), Diepenbeek, Belgium; ^8^Department of Neurology, School of Medicine, University of Ioannina, Ioannina, Greece; ^9^Laboratory of Molecular Genetics, Immunology Department, Hellenic Pasteur Institute, Athens, Greece; ^10^Department of Clinical Pharmacology, School of Medicine, Aristotle University of Thessaloniki, Thessaloniki, Greece

**Keywords:** COVID-19 pandemic, people with multiple sclerosis (PwMS), caregivers, quality of life (QoL), vaccination, information and communications technology (ICT) tools

## Abstract

**Background:** In the context of the coronavirus disease 2019 (COVID-19) pandemic, the constant needs of people with multiple sclerosis (PwMS) and their caregivers were urgently highlighted.

**Aim:** The present study aims to capture the effects of the COVID-19 pandemic in several aspects of the quality of life of PwMS, in perception and behavior to COVID-19 and multiple sclerosis (MS), as well as concerning healthcare, working conditions, and the willingness toward COVID-19 vaccination.

**Methods:** This study is an initiative of the Hellenic Academy of Neuroimmunology (HEL.A.NI.) and it has been included in the MS Data Alliance (MSDA) Catalog, which can be accessed after creating an account on https://msda.emif-catalogue.eu/login. Two online questionnaires were administered: (i) impact of the COVID-19 pandemic on the quality of life, behavior, and healthcare of PwMS (Questionnaire A) and (ii) vaccination against COVID-19 (Questionnaire B). People with MS were invited to participate by the Hellenic Federation of Persons with Multiple Sclerosis (HFoPwMS).

**Results:** Three-hundred-ninety PwMS responded to Questionnaire A, whereas 176 PwMS provided answers for Questionnaire B. Older age, longer disease duration, and higher MS-related disability were associated with the increased perceived sensitivity toward severe acute respiratory syndrome coronavirus 2 (SARS-CoV-2) infection, as well as the increased perceived severity of COVID-19 upon potential infection. A significant proportion of PwMS experienced restricted access to MS-related health professionals, disease-modifying therapy (DMT) prescription, and/or to MS-related laboratory examination due to the pandemic. Subgroups of PwMS reported exacerbated symptoms (i.e., chronic MS-related symptoms, fatigue and/or worsening of pre-existing fatigue, and sexual dysfunction and or/worsening of pre-existing sexual dysfunction). Overall, the majority of the participants reported either a strong willingness to get vaccinated against COVID-19 or a likeliness to undergo vaccination. Being aware of the HEL.A.NI. recommendations regarding COVID-19 vaccination for PwMS were reported to increase the willingness of the participants to receive the vaccine.

**Conclusions:** Our results highlight the necessity of scientific and patient organizations in taking joint action to increase awareness on health-related issues during the pandemic and to provide accurate and up-to-date guidance for PwMS. Online information and communications technology (ICT) tools for polling public belief and behavior may prove valuable as means of retaining active routes of communication between stakeholders.

## Introduction

In the context of the COVID-19 pandemic, the constant needs of people with multiple sclerosis (PwMS) and their caregivers were urgently highlighted, while ways of more effective management of these needs are extensively investigated (1, 2). Several scientific organizations are recommending alterations in the management of multiple sclerosis (MS) (3–5). These changes aim to minimize the direct interaction of patients with the health system, thus reducing the risk of exposure and infection with the severe acute respiratory syndrome coronavirus 2 (SARS-CoV-2) (6, 7). Effective ways of interacting (e.g., platforms and other telemedicine technologies) for PwMS and their caregivers with MS centers and the related facilities of public health are currently being investigated to allow for medical consultation services and disease management in the absence of physical presence (8–10). Moreover, the level of citizens' knowledge regarding SARS-CoV-2 and severe COVID-19 prevention measures is expected to affect their willingness to participate in vaccination programs against COVID-19, a critical step toward the control of the pandemic.

The COVID-19 pandemic and the social distancing measures have obvious negative effects on the self-reported psychological, mental, and social well-being, as well as the healthcare, of many PwMS (11, 12). MS is a complex multifactorial disease influenced by several environmental factors that may affect the immune system and the overall clinical course. Psychological distress may affect the disease and potentially alter its course and manifestations (12). In the context of the COVID-19 pandemic, inevitable measures of spreading prevention produce global social and economic alterations with a related psychological burden (13, 14), as indicated by studies on the impact of quarantine and the related coping mechanisms (15, 16). People with MS exhibit increased levels of COVID-19-related anxiety and this observation has been linked with an increased degree of commitment toward following personal preventative measures (17). However, as a proportion of PwMS may adopt dysfunctional coping strategies during stressful conditions, they may be more vulnerable to the stress-related harmful effects of the COVID-19 pandemic (14).

The use of online tools to assess the impact of the COVID-19 pandemic has been used since the first wave of the pandemic and significantly reflects the readiness of the scientific community to maintain channels of communication with society (9, 18). Recently, many worldwide scientific societies and patient organizations are turning to online information and communications technology (ICT) tools to capture the experience of health professionals in their effort to provide their services within the current crisis, as well as to depict, and possibly quantify, the degree of exposure of the society and the health system to the crisis (19, 20). These initiatives can reflect the action readiness levels of scientific organizations when addressing their registered members. However, the current legal framework regarding personal data and personal sensitive data protection and privacy sets clear pre-requisites to the scientific-medical community, to reach out directly to patients-citizens *via* online ICT tools. In the context of the COVID-19 pandemic, the development of online tools that directly assess the impact of the health crisis in the quality of life of patients with chronic diseases, such as MS, with a capacity to capture their experience regarding changes in the way they interact with the health system and MS centers, is particularly valuable (21, 22).

The present study aims to capture the effects of the COVID-19 pandemic in several aspects of the quality of life, perception, and behavior in relation to COVID-19 and MS, as well as in the healthcare, working conditions, and willingness toward COVID-19 vaccination of PwMS, by implementing two online available, self-administered questionnaires. Through our initiative, we aspire to improve the actions taken at the national level in response to the COVID-19 pandemic in relation to PwMS. Further, we aim to contribute to the effort of the global community to make appropriate decisions and implement protective strategies in response to the COVID-19 pandemic and possible future health crises.

## Methods

### The Study

This study is an initiative of the Hellenic Academy of Neuroimmunology (HEL.A.NI.) and the 2nd Neurological University Department of the Aristotle University of Thessaloniki (AUTH) in collaboration with the Laboratory of Medical Physics and Digital Innovation. The study received the approval of the Bioethics Committee of the School of Medicine of the AUTH (Approval Nr. 6322/29-7-2020). The HEL.A.NI. initiative has been included in the MS Data Alliance (MSDA) Catalog and the vaccination questionnaire was used in the mapping exercise of COVID-19 vaccine protocols recently performed by the MSDA to support the alignment in updating COVID-19 in MS surveys and data collections, thereby aiming to facilitate collaborative research between initiatives (23).

### Recruitment

Adult PwMS were invited to participate in the study by the Hellenic Federation of Persons with Multiple Sclerosis (HFoPwMS), an umbrella organization representing nine associations of patients with MS from Greece and recognized by the Athens Court of First Instance (Decision No. 6403/2009). The HFoPwMS is a member of the National Confederation of People with Disabilities (NCPD) and an associate member of the European Multiple Sclerosis Platform (EMSP). The submission of an e-mail address was required for the respondents to register for both questionnaires (A and B), to ensure the reliability of the answers. All data were anonymized prior to the analysis and the anonymization protocol to be followed was described to the participants in detail before they provided their online informed consent. Participants provided online informed consent prior to their participation and acknowledged the following: (i) the anonymized data will be stored permanently and published for possible later use by other scientists. Conclusions about the participants or other persons are not possible, (ii) the data will be treated in accordance with the regulations of the European general data protection regulation (GDPR EU), (iii) participation in the study is voluntary and can be terminated at any time and without providing a specific reason, and (iv) participation in the research does not expose the participants to any significant risk. For a detailed reference in the information sheet, the informed consent, and the GDPR agreement, see Annex 1.

People with MS registered with the HFoPwMS, who had previously provided consent to receive notifications by the HFoPwMS, received e-mail invitations to participate in the study (Questionnaire A) *via* the HEL.A.NI. website, on April 20, 2020, with weekly reminders sent until June 2020 and again in October and November 2020. Similarly, PwMS who were registered with the HFoPwMS received e-mail invitations to participate in the study's questionnaire B, on February 11, 2021, and weekly thereafter until May 2021. The participants followed the links on the HEL.A.NI. website and completed the surveys in a Google form in a cross-sectional design. The participants were considered to be enrolled at survey completion.

As stated in the Protocol Template of the COVID-19 Snapshot Monitoring (COSMO) Study, a sample size of *n* = 1,000 per wave is recommended to reach a sample representative of the native population in terms of demographic characteristics. However, as the present study refers to PwMS as participants, smaller sample size may be applicable. More specifically, the sample size estimation was conducted as recommended in (24, 25). The total number of PwMS in Greece was extracted by Bakirtzis et al. (26), approximately equal to 21.218 people (197.8/100.000 population). Based on this method, the sample size necessary for a valid conclusion for the PwMS in Greece was calculated to equal to *n* = 378 PwMS.

### Questionnaires

Two online, self-administered questionnaires were constructed.

#### Impact of the COVID-19 Pandemic on the Quality of Life, Behaviors, and Healthcare of PwMS (Questionnaire A)

Questionnaire A is an adaptation from the COSMO study (27), that includes critical factors for behavioral change in the population to avoid the transmission of COVID-19 (i.e., risk perceptions, trust, use of information sources, knowledge, as well as barriers, and drivers to recommended behaviors). Further adaptation for the MS population was achieved by the addition of several items regarding their demographics, MS clinical characteristics, MS healthcare, and occupational status. Questionnaire A is available in a Google Forms environment *via* the website of HEL.A.NI. in Greek (http://www.helani.gr/) and English (http://en.helani.gr/). For the introduction page of the English version of Questionnaire A and the related privacy notice and consent declaration, see Annex 1.

In the protocol template of the COSMO study (27), it is stated that an *ad hoc* approach was applied for the review and validation of the original questionnaire, due to the urgency of the need for data collection. The cognitive and pilot testing of the questionnaire by the research group of the present study or more in-depth processes to culturally adapt the questionnaire (28) were hindered for reasons related to quarantine measures that prevented in-depth communication between health professionals and the participants, as well as to time restriction due to the urgency of the pandemic and the associated burden in the health services.

However, the reliability of Questionnaire A was assessed based on its internal structure (29). Part 1 evaluated mainly descriptive clinical information and demographics. Parts 2 and 3 exhibited satisfactory internal consistency with Cronbach's Alpha value of ≥0.7. Especially Part C, referring to the experience of PwMS in relation to COVID-19 exhibited a high internal consistency (for Part 2 Cronbach's Alpha value 0.731 and for Part 3 Cronbach's Alpha value 0.9). Moreover, special effort was employed for the translation of the Questionnaire. The translation was conducted by the members of the research group of the present study, researchers, and health professionals with long-term experience in MS management and the validation of tools for MS neurocognitive evaluation and other MS-related outcomes (30, 31). Back-translation was used to document potential equivalence issues in selected items.

The approximate completion time for questionnaire A was estimated to be 60 min. Questionnaire A was divided into three parts to facilitate convenience to the respondents: **Part 1**: general information regarding PwMS (~15 min). **Part 2**: beliefs and daily behaviors regarding SARS-CoV-2 (~25 min). **Part 3**: beliefs regarding SARS-CoV-2 in relation to MS and the effect of the COVID-19 pandemic on disease management (~20 min). The answers to the separate three parts were unified at a later step of the quality control based on the common e-mail address.

#### Vaccination Against COVID-19 (Questionnaire B)

Questionnaire B aimed to capture the degree of information and the willingness of PwMS to be vaccinated against the coronavirus Sars-CoV-2. The total time to complete the questionnaire is estimated to be ~10 min. For the translation of the introduction page, Questionnaire B, and the related privacy notice and consent declaration, see Annex 2.

### Data Analysis

Though both questionnaires targeted PwMS *via* the aforementioned organizations and associations (please see section Recruitment), they were also designed to easily identify non-MS respondents and these participations were excluded. Before the data anonymization and the subsequent data analysis, a step of quality control took place. Specifically, double entries, which could be identified by the email addresses, were removed. Variables were reported as means ± SE, or as percentages, as appropriate. There were no pre-specified hypotheses. The statistical significance was set at α = 0.05. All analyses were performed by the use of IBM^®^ SPSS^®^ Statistics software (27.0) (IBM, Armonk, New York, United States).

## Results

### Impact of the COVID-19 Pandemic on the Quality of Life, Behaviors, and Healthcare of PwMS (Questionnaire A)

#### Respondents

At the time of the survey administration, HFoPwMS listed ~4,500 members, accounting for the members of the contributing associations of patients. Before quality control, 442 answers were received for Questionnaire A-Part 1, 302 answers were received for Questionnaire A-Part 2, and 305 answers were received for Questionnaire A-Part 3. Following the quality control and removal of double entries, 390 answers were considered for Part 1, 293 answers for Part 2, and 305 answers for Part 3 of Questionnaire A, (response rate 8.67, 6.51, and 6.78%, respectively). This percentage is comparable to previous studies in the field (32). Of note, all participants that answered Part 2 also answered Part 1 of Questionnaire A. However, of the 305 participants who answered Part 3, only 274 also answered Part 1, therefore, the data concerning age and gender in the respective tables are presented only for those 274 participants. The male/female numbers of participants for the three Parts were 105/285, 77/216, and 72/202, respectively.

Concerning the time-points of participation, the majority of answers were received in the period April–May 2020, with fewer answers received in June–July–August 2020. Following the reminders sent from the HFoPwMS, additional answers were received in October–November–December 2020, but these answers were for Questionnaire A-Part 3, focusing on COVID-19 and MS. Therefore, the distribution of answers for the three parts of Questionnaire A across time-points was as follows: Part 1 April–May 2020 (351 answers), June–July–August 2020 (33 answers), October–November–December 2020 (5 answers); Part 2 April–May 2020 (266 answers), June–July–August 2020 (21 answers), October–November–December 2020 (6 answers); and Part 3 April–May 2020 (248 answers), June–July–August 2020 (20 answers), October–November–December 2020 (35 answers). Since the number of answers was considerably uneven across time-points for all three parts of Questionnaire A, comparative analysis across the time-points was not conducted in the overall study setting, as it was unlikely to yield safe conclusions. Few exploratory comparisons, only concerning the perceptions and behaviors of the participants in relation to COVID-19, based on the answers in Part 2, were conducted between the time-points April–May 2020 (initial phase of the pandemic) and the pooled June–December 2020 (subsequent phases of the pandemic). The results are presented taking into account limitations with respect to an uneven number of answers between time points.

#### Demographics

**Figure 1:**
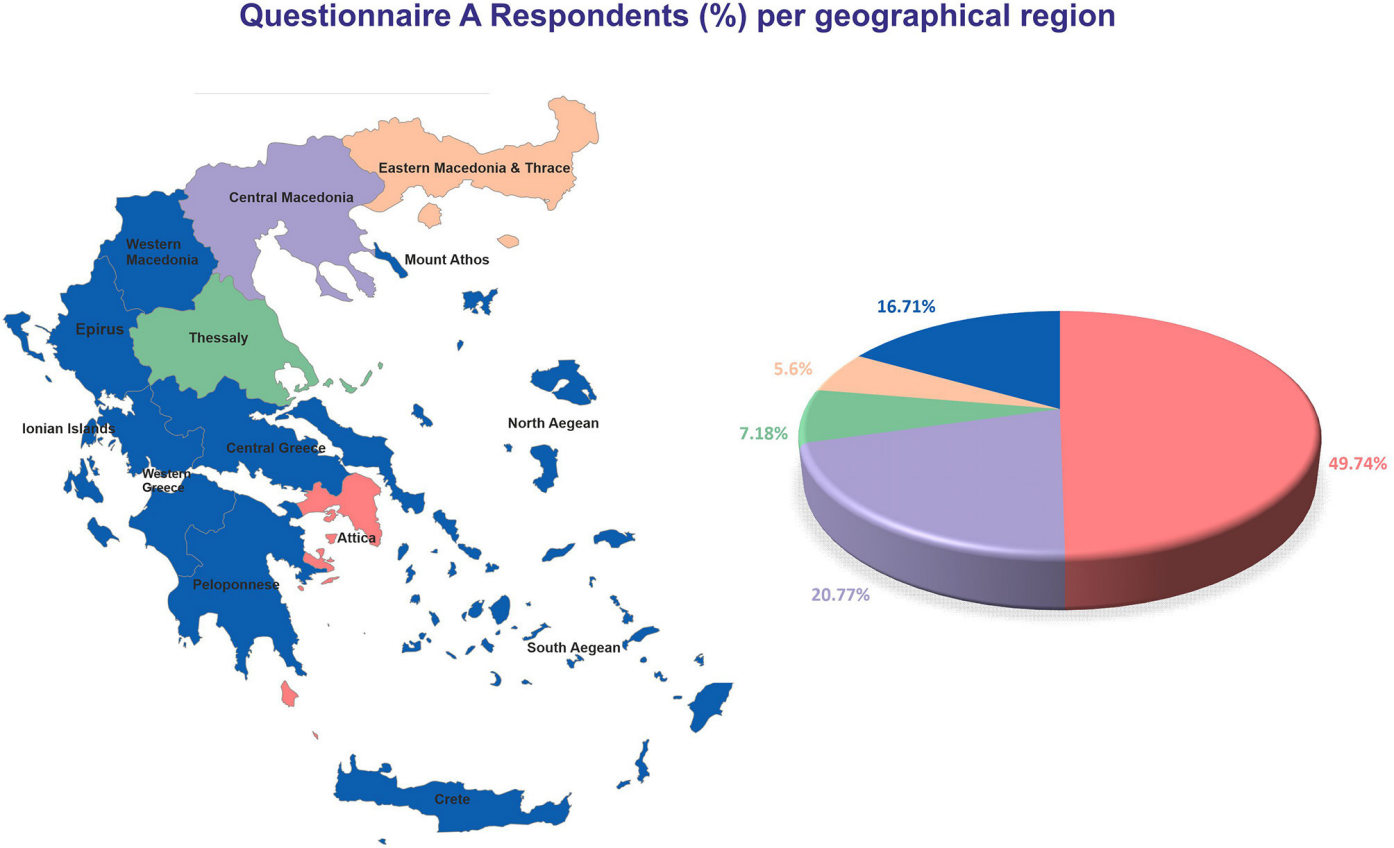
Geographical distribution of respondents for Questionnaire A.

The average age of the respondents was 45.4 ± 1.03 for men (*N* = 105, 26.92%) and 43.99 ± 0.65 for women (*N* = 285, 73.08%) (*p* = 0.253; Table 1). Regarding school education, the largest group represented among the participants were university graduates (*N* = 187, 47.95%). Few participants were health professionals (*N* = 43, 11.03%). The higher percentage of participants were living in Attica (*N* = 194, 49.74%), followed by Central Macedonia (*N* = 81, 20.77%), Thessaly (*N* = 28, 7.18%), and Eastern Macedonia and Thrace (*N* = 22, 5.6%), whereas the rest of the participants (*N* = 65, 16.71%) were distributed across the remaining geographical parts of Greece (13 in total) (Figure 1). One hundred sixty-one participants (41.28%) were smokers.

**Table 1:** Participant demographics.

**Participants' gender**	**Male**				**Female**			
**Participants' age (years)**	**≤ =55**	**%**	**>55**	**%**	**≤55**	**%**	**>55**	**%**
*n* Participants (% of 390)	88	22.56	17	4.36	246	63.08	39	10.00
**School education** ***n*** **(%)**								
Elementary school (up to 6 years of schooling)	0	0.00	0	0.00	0	0.00	0	0.00
At least 9 years	3	0.77	2	0.51	0	0.00	1	0.26
At least 12 years	35	8.97	4	1.03	66	16.92	11	2.82
Technical Institute	13	3.33	2	0.51	52	13.33	6	1.54
University	37	9.49	9	2.31	121	31.03	20	5.13
**Health care professional** ***n*** **(%)**								
Yes	9	2.31	1	0.26	32	8.21	1	0.26
No	79	20.26	16	4.10	214	54.87	38	9.74
**Smoking** ***n*** **(%)**								
Yes	38	9.74	3	0.77	103	26.41	17	4.36
No	49	12.56	14	3.59	143	36.67	21	5.38
Unsure	1	0.26	0	0.00	0	0.00	1	0.26
**Geographical area of living** *n* (%)								
Western Greece	1	0.26	1	0.26	6	1.54	1	0.26
Epirus	0	0.00	0	0.00	1	0.26	0	0.00
Thessaly	5	1.28	2	0.51	19	4.87	2	0.51
Eastern Macedonia and Thrace	4	1.03	0	0.00	17	4.36	1	0.26
Crete	1	0.26	0	0.00	5	1.28	0	0.00
Central Macedonia	20	5.13	3	0.77	55	14.10	3	0.77
Western Macedonia	5	1.28	0	0.00	7	1.79	0	0.00
North Aegean	3	0.77	0	0.00	6	1.54	2	0.51
South Aegean	1	0.26	0	0.00	3	0.77	2	0.51
Ionian Islands	2	0.51	0	0.00	2	0.51	0	0.00
Peloponnese	3	0.77	1	0.26	3	0.77	1	0.26
Attica	39	10.00	10	2.56	119	30.51	26	6.67
Central Greece	3	0.77	0	0.00	2	0.51	1	0.26

#### MS-Related Medical History

**Figure 2:**
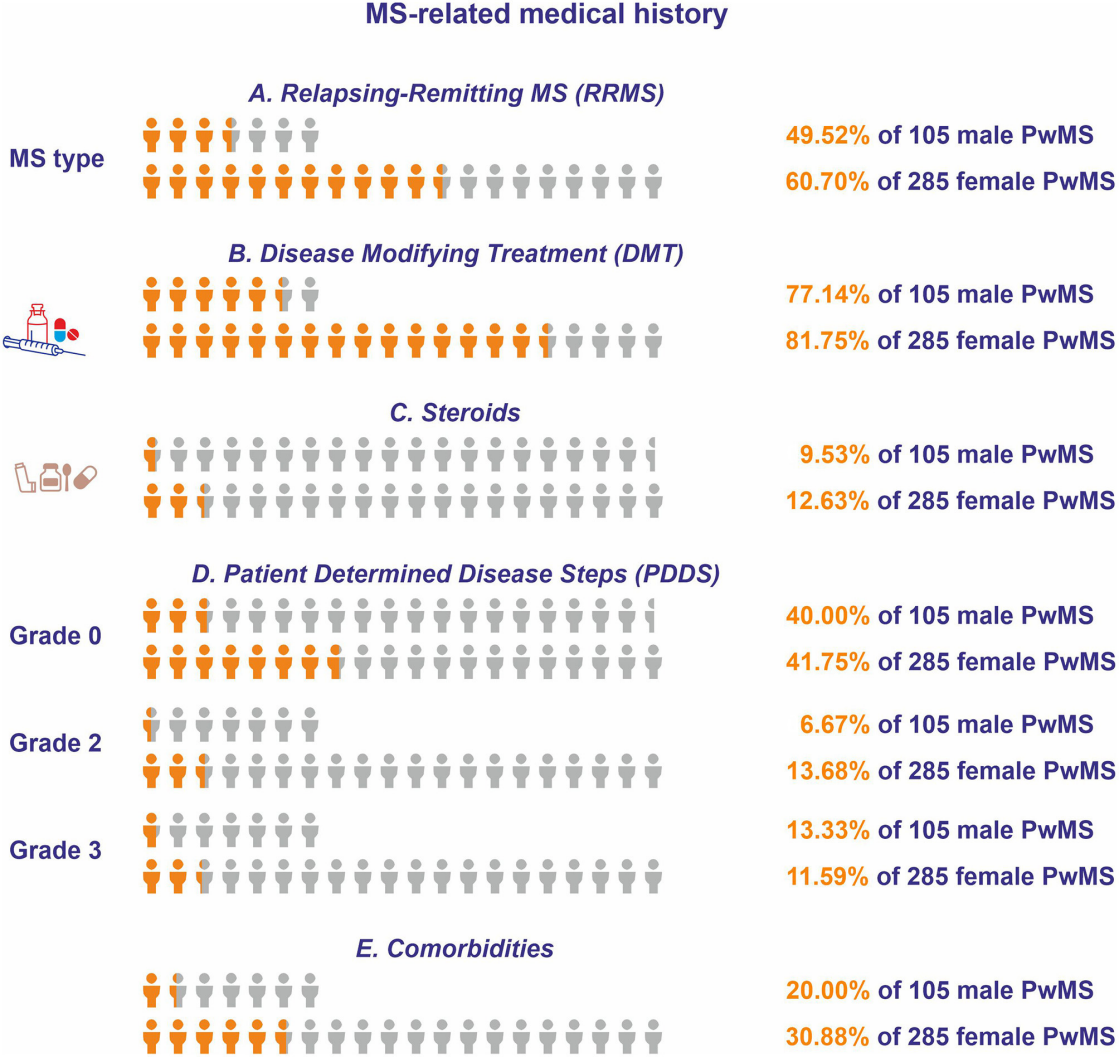
Multiple sclerosis-related medical histories for respondents of Questionnaire A. The orange icon of the human form depicts the portion of the male and female respondents who **(A)** had relapsing-remitting form of MS (RRMS), **(B)** received disease-modifying therapy (DMT), **(C)** received steroids during the 2 months prior to participation, **(D)** had patient determined disease steps (PDDS) of grade 0, grade 2, or grade 3, and **(E)** reported comorbidities. The light gray icon depicts the total number of male and female people with MS (PwMS).

On average, the disease duration was 14.34 ± 0.99 years for men (*N* = 98) and 14.71 ± 0.64 for women (*N* = 273) (*p* = 0.759; Table 2). The MS diagnosis occurred 11.3 ± 0.85 years prior to the participation in the study for men (*N* = 102), and 12.08 ± 0.59 years prior to the participation in the study for women (*N* = 283). The majority of participants were exhibiting a relapsing-remitting form of MS (RRMS) (men: *N* = 52, 49.52%; women: *N* = 173, 60.7%) (Figure 2A). Eighty-one (77.14%) of male participants and 233 (81.75%) of female participants received disease-modifying treatment (DMT) (Figure 2B).

**Table 2:** Clinical characteristics of participants.

**Participants' gender**	**Male**				**Female**			
**Participants' age (years)**	**≤55**	**%**	**>55**	**%**	**≤55**	**%**	**>55**	**%**
*n* Participants (% of 390)	88	22.56	17	4.36	246	63.08	39	10.00
**Disease duration** *Mean ± SE*	12.6 ± 0.89		23.25 ± 3.3		13.19 ± 0.65		25.06 ± 1.4	
**Years from diagnosis** *Mean ± SE*	10.15 ± 0.86		17.5 ± 2.33		10.76 ± 0.6		20.63 ± 1.5	
**Type of MS** ***n*** **(%)**								
Relapsing—remitting	46	11.79	6	1.54	158	40.51	15	3.85
Secondary progressive	13	3.33	7	1.79	27	6.92	12	3.08
Primarily progressive	10	2.56	1	0.26	11	2.82	3	0.77
I don't know/don't answer	19	4.87	3	0.77	50	12.82	9	2.31
**Currently receiving DMT** ***n*** **(%)**								
Yes	71	18.21	10	2.56	211	54.10	22	5.64
No	17	4.36	7	1.79	29	7.44	17	4.36
**Type of current DMT** *n* (%)								
Interferon-β	18	4.62	3	0.77	45	11.54	5	1.28
Glatiramer acetate	6	1.54	1	0.26	25	6.41	5	1.28
Dimethyl fumarate	20	5.13	2	0.51	49	12.56	5	1.28
Teriflunomide	5	1.28	0	0.00	16	4.10	2	0.51
Natalizumab	3	0.77	0	0.00	13	3.33	0	0.00
Fingolimod	10	2.56	2	0.51	44	11.28	2	0.51
Alemtuzumab	2	0.51	1	0.26	2	0.51	0	0.00
Cladribine	0	0.00	0	0.00	3	0.77	0	0.00
Anti-CD20	3	0.77	0	0.00	6	1.54	0	0.00
I don't know/don't answer	4	1.03	1	0.26	8	2.05	3	0.77
**Previously receiving DMT** ***n*** **(%)**								
Yes	47	12.05	13	3.33	160	41.03	21	5.38
No	41	10.51	4	1.03	84	21.54	17	4.36
**Type of previous DMT** ***n*** **(%)**								
Interferon-β	33	8.46	12	3.08	120	30.77	16	4.10
Glatiramer acetate	12	3.08	6	1.54	46	11.79	8	2.05
Dimethyl fumarate	7	1.79	0	0.00	16	4.10	3	0.77
Teriflunomide	5	1.28	1	0.26	13	3.33	3	0.77
Natalizumab	4	1.03	3	0.77	23	5.90	7	1.79
Fingolimod	6	1.54	1	0.26	24	6.15	6	1.54
Alemtuzumab	1	0.26	0	0.00	8	2.05	0	0.00
Cladribine	1	0.26	0	0.00	4	1.03	0	0.00
anti-CD20	0	0.00	0	0.00	5	1.28	0	0.00
I don't know/don't answer	0	0.00	0	0.00	0	0.00	0	0.00
**Steroids in the last 2 months** ***n*** **(%)**								
Yes	10	2.56	0	0.00	32	8.21	4	1.03
No	78	20.00	17	4.36	214	54.87	35	8.97
I don't know/don't answer	0	0.00	0	0.00	0	0.00	0	0.00
**PDSS** ***n*** **(%)**								
Normal	40	10.26	2	0.51	112	28.72	7	1.79
Mild disability	7	1.79	2	0.51	27	6.92	1	0.26
Moderate disability	6	1.54	1	0.26	37	9.49	2	0.51
Gait disability	10	2.56	4	1.03	28	7.18	5	1.28
Early cane	6	1.54	3	0.77	17	4.36	5	1.28
Late cane	7	1.79	2	0.51	15	3.85	8	2.05
Bilateral support	6	1.54	1	0.26	6	1.54	7	1.79
Wheelchair/scooter	4	1.03	1	0.26	3	0.77	3	0.77
Bedridden	2	0.51	1	0.26	1	0.26	1	0.26
**Comorbidities** ***n*** **(%)**								
Yes	18	4.62	3	0.77	68	17.44	20	5.13
No	64	16.41	13	3.33	159	40.77	19	4.87
I don't know/don't answer	6	1.54	1	0.26	19	4.87	0	0.00
**Type of comorbidity** ***n*** **(%)**								
Autoimmune thyroid disease	3	0.77	0	0.00	46	11.79	4	1.03
Depression	5	1.28	1	0.26	10	2.56	7	1.79
Hypertension	4	1.03	2	0.51	2	0.51	4	1.03
Other cardiovasular disease	4	1.03	0	0.00	1	0.26	0	0.00
Diabetes	5	1.28	0	0.00	10	2.56	5	1.28
Inflammatory bowel disease	2	0.51	0	0.00	4	1.03	0	0.00
Psoriasis	2	0.51	0	0.00	7	1.79	0	0.00
Non-identified rheumatological disease	2	0.51	0	0.00	5	1.28	0	0.00
Sjogren syndrome	0	0.00	0	0.00	1	0.26	0	0.00
Anti-phospholipid syndrome	0	0.00	0	0.00	1	0.26	0	0.00
Ankylosing spondylitis	0	0.00	0	0.00	2	0.51	0	0.00
Coeliac disease	0	0.00	0	0.00	1	0.26	0	0.00
Vitiligo	0	0.00	0	0.00	2	0.51	0	0.00
Epilepsy	0	0.00	0	0.00	1	0.26	0	0.00

*MS, Multiple Sclerosis; DMT, Disease-modifying Treatment; PDDS, Patient Determined Disease Steps*.

The highest percentage of participants treated by DMT received dimethyl fumarate (men: *N* = 22, 27.16%; women: *N* = 54, 23.18), followed by interferon-β (men: *N* = 21, 25.92%; women: *N* = 50, 21.46%), and by fingolimod (men: *N* = 12, 14.81%; women: *N* = 46, 19.74%). With respect to the relative frequency of first- vs. second-line DMTs, 207 (53.08%) participants reportedly received first-line DMTs, whereas 74 (18.97%) participants received second-line DMTs. Of the 207 participants who were under first-line DMTs at the time of their participation to the study, 108 (52.17%) received injectables, whereas 99 (47.82%) received oral agents (Table 2). Moreover, the majority of participants had received another DMT in the past (men: *N* = 60, 57.14%; women: *N* = 181, 63.5%). With respect to the previously received DMT, the majority received interferon-β (men: *N* = 45, 75%; women: *N* = 136, 75.14%) followed by glatiramer acetate (men: *N* = 18, 30%; women: *N* = 54, 29.83%). The main reasons for DMT discontinuation for those currently free of DMT were treatment inefficacy (men: *N* = 5, 20.83%; women: *N* = 46, 100%), adverse events (men: *N* = 3, 12.5%; women: *N* = 18, 39.13%), and pregnancy planning (*N* = 18, 39.13%) (Table 2).

Forty-six (11.8%) participants (men: *N* = 10, 9.53%; women: *N* = 36, 12.63%) received steroids at some point during the 2 months prior to participation, a time frame that in all cases was after the onset of the pandemic (Figure 2C). The patient determined disease steps (PDDS) was normal (grade = 0) for the majority of participants (men: *N* = 42, 40%; women: *N* = 119, 41.75%), followed by gait disability (grade = 3) (men: *N* = 14, 13.33%; women: *N* = 33, 11.59%), and by moderate disability (grade = 2) (men: *N* = 7, 6.67%; women: *N* = 39, 13.68%) (Figure 2D). One-hundred-nine participants reported the existence of comorbidities (men: *N* = 21, 20%; women: *N* = 88, 30.88%) (Figure 2E). The most commonly reported comorbidity was autoimmune thyroid disease (men: *N* = 3, 2.9%; women: *N* = 50, 17.54%), followed by depression (men: *N* = 6, 5.7%; women: *N* = 17, 6%) and diabetes (men: *N* = 5, 4.8%; women: *N* = 15, 5.26%).

#### SARS-CoV-2 Exposure

Three female PwMS under the age of 55 (mean age 37.33 ± 3.18) were tested to be *SARS-CoV-2* positive (Table 3). Two patients lived in Attica and one patient lived in Central Macedonia. One patient received dimethyl fumarate for RRMS, did not report steroid treatment during the last 2 months prior to participation, and reported a PDDS of grade 1. The patient did not require hospitalization and the infection was fully resolved. The second patient also received dimethyl fumarate for RRMS, did not report steroid treatment during the last 2 months prior to participation, and reported a PDDS of grade 2. The patient did not require hospitalization and the infection was fully resolved. Another patient reported secondary progressive MS (SPMS) and status free of DMT. None of the female PwMS who tested positive for SARS-CoV-2 reported steroid treatment and/or hospitalization and the infection was fully resolved. The disease duration was 1 and 4 years for the patients with RRMS, whereas it was 16 years for the patient with SPMS. With respect to comorbidities, one patient also reported autoimmune thyroid disease and one patient reported inflammatory bowel disease. Twenty-six patients (8.7%) reported knowing someone exposed to SARS-CoV-2. The patients who reported knowing someone who tested SARS-CoV-2 positive were of comparable age (44.54 ± 2.03 vs. 43.99 ± 0.66, *p* = 0.81) and reported comparable levels of SARS-CoV-2 pandemic-related anxiety (3.35 ± 0.37 vs. 3.2 ± 0.11, *p* = 0.685), compared with patients who did not know someone exposed to SARS-CoV-2, respectively. Moreover, they did not consider that they were under a higher probability of SARS-CoV-2 infection (4.15 ± 0.38 vs. 4.21 ± 0.09, *p* = 0.889).

**Table 3:** COVID-19-related behavior and beliefs.

**Participants' gender**	**Male**					**Female**				
**Participants' age (years)**	**≤55**	**% or SE**	**>55**	**% or SE**	***p***	**≤55**	**% or SE**	**>55**	**% or SE**	***p***
*n* Participants (% of 293)	65	22.18	12	4.10		189	64.51	27	9.22	
**SARS-CoV-2 positive** ***(n, %)***
Yes	0	0.00	0	0.00	0.80	3	1.02	0	0.00	0.633
No	56	19.11	10	3.41		157	53.58	25	8.53	
Unsure	9	3.07	2	0.68		29	9.90	2	0.68	
**Know someone exposed to SARS-CoV-2** ***(n, %)***
Yes	4	1.37	1	0.34	0.70	16	5.46	3	1.02	0.858
No	61	20.82	11	3.75		173	59.04	24	8.19	
**COVID-19 level of knowledge** *(mean, SE)*	5.11	0.17	5.42	0.29	0.452	5.41	0.08	5.70	0.18	0.199
**Infection prevention knowledge** *(mean, SE)*	5.52	0.16	5.67	0.26	0.720	5.94	0.07	6.04	0.19	0.642
**Perceived risk of infection** *(mean, SE)*	3.92	0.22	4.58	0.47	0.233	4.32	0.11	4.07	0.36	0.524
**Perceived severity upon potential infection** *(mean, SE)*	5.25	0.21	6.00	0.33	0.140	5.38	0.10	5.78	0.29	0.171
**Perceived sensitivity** *(mean, SE)*	5.15	0.22	5.42	0.43	0.631	5.29	0.10	5.96	0.28	**0.021**
**Knowledge regarding protection measures** *(mean, SE)*	5.88	0.11	6.00	0.21	0.660	6.05	0.07	6.00	0.18	0.802
**Importance of avoidance of infection** *(mean, SE)*	4.45	0.17	5.00	0.30	0.191	4.57	0.09	4.44	0.23	0.643
**Self-quarantine willingness** *(mean, SE)*	4.69	0.24	5.00	0.39	0.508	4.60	0.14	4.85	0.37	0.532
**SARS-CoV-2 infection possibility (distance)** *(mean, SE)*	4.15	0.20	4.50	0.19	0.228	4.32	0.10	4.63	0.33	0.385
**SARS-CoV-2 infection spread** *(mean, SE)*	5.22	0.20	4.83	0.27	0.269	4.99	0.10	5.15	0.36	0.685
**SARS-CoV-2 infection frequent thinking** *(mean, SE)*	3.92	0.20	4.00	0.39	0.874	3.90	0.11	4.11	0.37	0.513
**SARS-CoV-2 infection fear** *(mean, SE)*	4.43	0.23	3.58	0.53	0.155	3.63	0.13	3.67	0.36	0.920
**SARS-CoV-2 infection worry** *(mean, SE)*	3.51	0.22	2.83	0.27	0.065	3.17	0.13	2.96	0.36	0.577
**SARS-CoV-2 infection helpless/powerful** *(mean, SE)*	3.94	0.22	3.42	0.36	0.334	3.75	0.13	3.41	0.36	0.357
**SARS-CoV-2 infection stress** *(mean, SE)*	3.91	0.23	3.50	0.48	0.483	3.47	0.13	3.52	0.39	0.888
**Source of COVID-19-related information level of trust** ***(mean, SE)***										
Public television channels	3.35	0.23	4.67	0.51	**0.026**	3.62	0.12	4.56	0.37	**0.010**
Newspapers	3.09	0.19	3.92	0.45	**0.098**	3.48	0.11	4.22	0.31	**0.016**
Family	3.58	0.21	4.25	0.49	0.212	3.62	0.11	3.74	0.34	0.695
Colleagues	3.58	0.20	3.83	0.41	0.614	3.60	0.11	3.59	0.36	0.974
Health professionals	5.46	0.19	6.00	0.28	0.124	5.63	0.09	5.59	0.32	0.878
Private television channels	2.92	0.20	3.75	0.55	0.121	3.04	0.12	3.52	0.31	0.156
Online sites	3.29	0.20	2.67	0.40	0.220	3.61	0.11	3.63	0.33	0.948
Magazines	2.68	0.18	2.75	0.33	0.870	2.82	0.10	2.81	0.24	0.985
Social media	2.74	0.21	2.17	0.30	0.127	2.81	0.11	2.26	0.24	**0.062**
Online information	3.18	0.19	3.17	0.44	0.970	3.70	0.11	3.22	0.33	0.133
Private radio stations	2.82	0.18	3.25	0.30	0.334	3.05	0.11	3.00	0.30	0.879
Public radio stations	3.15	0.21	3.58	0.48	0.427	3.21	0.12	3.89	0.34	0.042
**Type of COVID-19-related information frequently sought** ***(n, %)***										
Symptoms of infection	55	18.77	12	4.10	0.145	167	57.00	22	7.51	0.312
Personal stories	27	9.22	5	1.71	0.993	80	27.30	11	3.75	0.876
Vaccine development	56	19.11	12	4.10	0.170	164	55.97	23	7.85	0.821
Treatment development	58	19.80	12	4.10	0.233	176	60.07	25	8.53	0.919
Personal measures for limiting virus spread	54	18.43	11	3.75	0.451	170	58.02	24	8.19	0.865
Caregiving measures for susceptible persons	43	14.68	11	3.75	0.076	152	51.88	19	6.48	0.229
Children's education related to school behavior	42	14.33	5	1.71	0.134	110	37.54	9	3.07	**0.015**
Travel limitations due to the pandemic	38	12.97	8	2.73	0.594	114	38.91	22	7.51	**0.033**

*COVID-19, coronavirus disease of 2019; SARS-CoV-2, Severe Acute Respiratory Syndrome Coronavirus-2; SE, Standard Error of mean. Bold values indicate p < 0.1*.

#### COVID-19 Awareness

The PwMS that completed Part 2 of Questionnaire A (*N* = 293) were aware of the pandemic before they participated in the study. Overall, the female PwMS, in contrast to males, tended to estimate both the degree of their knowledge on SARS-CoV-2 (5.45 ± 0.07 vs. 5.16 ± 0.15, *p* = 0.057, respectively) and the degree of their knowledge on SARS-CoV-2 spreading prevention measures (5.95 ± 0.07 vs. 5.55 ± 0.14, *p* = 0.012, respectively) higher. Conversely, men reported higher levels of SARS-CoV-2-related fear compared to women (3.64 ± 0.12 vs. 4.3 ± 0.22, *p* = 0.006; a higher score denotes a lower degree of self-reported fear for this question). There was no gender difference with respect to the perceived degree of sensitivity toward SARS-CoV-2 infection (5.19 ± 0.19 vs. 5.38 ± 0.1, *p* = 0.412). However, female PwMS older than 55 years reported an increased perceived degree of sensitivity toward SARS-CoV-2 infection compared with 55-year-old or younger women (5.95 ± 0.28 vs. 5.29 ± 0.1, *p* = 0.021). This effect was not evident for men (5.42 ± 0.43 vs. 5.15 ± 0.22, *p* = 0.631) (Table 3).

On a gender-stratified analysis, the age of the female PwMS was significantly correlated with both the perceived COVID-19 severity upon SARS-CoV-2 infection (*r* = 0.244, *p* < 0.001) and the perceived degree of sensitivity toward SARS-CoV-2 infection (*r* = 0.243, *p* < 0.001). Moreover, age was correlated with the degree of feeling helpless concerning preventing SARS-CoV-2 infection (*r* = 0.196, *p* = 0.004). The disease duration of the female PwMS was significantly correlated with both the perceived COVID-19 severity upon SARS-CoV-2 infection (*r* = 0.217, *p* = 0.002) and the perceived degree of sensitivity toward SARS-CoV-2 infection (*r* = 0.137, *p* = 0.048). Additionally, the self-reported disability (PDDS score) was correlated with both the perceived COVID-19 severity upon SARS-CoV-2 infection (*r* = 0.194, *p* = 0.004) and the perceived degree of sensitivity toward SARS-CoV-2 infection (*r* = 0.191, *p* = 0.005). The age of the male PwMS was significantly correlated with the perceived COVID-19 severity upon SARS-CoV-2 infection (*r* = 0.352, *p* = 0.002). Also, age was correlated to the degree of SARS-CoV-2-related fear (*r* = 0.299, *p* = 0.008) and worry (*r* = 0.226, *p* = 0.049) in the male but not in female PwMS. The disease duration of the male PwMS was significantly correlated with both the perceived COVID-19 severity upon SARS-CoV-2 infection (*r* = 0.263, *p* = 0.027) and the perceived degree of sensitivity toward SARS-CoV-2 infection (*r* = 0.274, *p* = 0.021). Moreover, self-reported disability (PDDS score) correlated with both the perceived COVID-19 severity upon SARS-CoV-2 infection (*r* = 0.458, *p* < 0.001) and the perceived degree of sensitivity toward SARS-CoV-2 infection (*r* = 0.333, *p* = 0.003). The type of MS did not seem to affect COVID-related awareness and/or the perceptions and worries of the participants with respect to the pandemic (data not shown). Similarly, depression did not seem to correlate with alterations in disease awareness and/or participants' perceptions and worries with respect to the pandemic (data not shown).

Both male and female PwMS older than 55 years old reported a higher level of trust for COVID-19-related information from public TV channels compared with PwMS of 55 years old or younger, respectively (men: 4.67 ± 0.51 vs. 3.35 ± 0.23, *p* = 0.026; women: 4.56 ± 0.37 vs. 3.62 ± 0.12, *p* = 0.01). Similarly, both male and female PwMS older than 55 years old reported higher level, or a tendency for a higher level of trust for COVID-19-related information from newspapers compared with PwMS of 55 years old or younger, respectively (men: 3.92 ± 0.45 vs. 3.09 ± 0.19, *p* = 0.098; women: 4.22 ± 0.31 vs. 3.48 ± 0.11, *p* = 0.016). Conversely, 55-year-old or younger female PwMS tended to have a higher level of trust for COVID-19-related information from social media compared with PwMS older than 55 years old (2.81 ± 0.11 vs. 2.26 ± 0.24, *p* = 0.062) but this was not seen in male PwMS. With respect to the type of COVID-19-related information frequently sought, symptoms of infection (53.2%), vaccine development (53%), treatment development (56.2%), personal measures for limiting virus spread (53.8%), and caregiving measures for susceptible persons (46.8%) were the most common topics (Table 3).

Upon few exploratory comparisons of COVID-19 awareness between the answers provided in two periods, namely, April–May 2020 (initial phase of the pandemic) and the pooled June–December 2020 (subsequent phases of the pandemic), the received answers did not exhibit significantly different results with respect to overall COVID-19 level of knowledge, the knowledge regarding infection prevention, the perceived risk of infection, the perceived severity upon infection, the perceived sensitivity upon infection, the knowledge regarding protection measures, the importance of avoidance of infection, the willingness to self-quarantine, as well as perceptions regarding SARS-CoV- 2 infection possibility, the infection spread speed, the frequency of thinking of the pandemic, the related fear, worry, helplessness and stress (data not shown).

#### COVID-19 and MS: Awareness and Concerns

With respect to the level of trust toward the sources of COVID-19-and-MS-related information, the treating Neurologist/MS specialist was the most trusted source of information (5.64 ± 0.09) followed closely by websites of MS-related scientific organizations (e.g., Hellenic Neurological Society, HEL.A.NI, etc.) (4.99 ± 0.11). Social media was among the least trusted sources of COVID-19-and-MS-related information (3.53 ± 0.12). Though 55 year old or younger male PwMS trusted this source more than men older than 55 years old (3.53 ± 0.27 vs. 2 ± 0.34, *p* = 0.001), such an effect was not observed for women (data stratification with respect to gender and age is presented in Table 4 for 274 participants who participated in Part 1 and 3 of the Questionnaire). The most frequently sought type of COVID-19-and-MS-related information regarded MS and infection-susceptible (“high-risk”) populations (93.8%), as well as the SARS-CoV-2 infection risk for PwMS on DMTs (88.32%). In contrast, the least frequently sought type of information was reports of pandemic-related personal stories of other PwMS (52.92%).

**Table 4:** COVID-19 and multiple sclerosis awareness.

**Participants' gender**	**Male**					**Female**				
**Participants' age (years)**	**≤55**	**% or SE**	**>55**	**% or SE**	***p***	**≤55**	**% or SE**	**>55**	**% or SE**	***p***
*n* of Participants (% of 274)	57	20.80	15	5.47		174	63.50	28	10.22	
**Level of trust toward sources of COVID and MS-related information** ***(mean, SE)***										
Treating neurologist	5.51	0.23	5.40	0.48	0.832	5.67	0.11	5.46	0.32	0.508
Sites of scientific organizations	4.72	0.25	4.47	0.52	0.647	5.07	0.13	5.00	0.42	0.877
MS center telephone communicational	3.37	0.27	2.93	0.57	0.469	2.93	0.16	3.82	0.38	**0.038**
MS center site	4.04	0.27	3.60	0.51	0.456	4.36	0.15	4.50	0.41	0.725
Other sites	3.93	0.23	3.07	0.40	0.080	4.29	0.14	4.29	0.42	0.997
Health professional consultation	4.09	0.27	4.40	0.48	0.595	4.13	0.16	4.61	0.39	0.273
Patients' organizations	4.16	0.28	3.80	0.52	0.553	3.92	0.16	4.75	0.40	**0.055**
Other PwMS	3.09	0.28	2.80	0.43	0.626	2.76	0.15	3.68	0.44	**0.057**
Social media	3.53	0.27	2.00	0.34	**0.001**	3.60	0.16	3.54	0.40	0.883
**Type of COVID and MS-related information frequently sought (** ***n*** **, %)**										
Risk of SARS-CoV infection for PwMS free from DMTs	35	12.77	9	3.28	0.921	87	31.75	16	5.84	0.483
Risk of SARS-CoV infection for PwMS under DMTs	50	18.25	12	4.38	0.442	162	59.12	18	6.57	**<0.001**
MS and working conditions during the pandemic	42	15.33	11	4.01	0.978	135	49.27	15	5.47	**0.025**
MS during the pandemic	55	20.07	15	5.47	0.462	162	59.12	25	9.12	0.765
DMTs for MS during the pandemic	50	18.25	12	4.38	0.442	150	54.74	17	6.20	**0.004**
Personal experience of PwMS regarding the pandemic	33	12.04	5	1.82	**0.090**	95	34.67	12	4.38	0.505
Caregiving for PwMS during the pandemic	33	12.04	10	3.65	0.538	95	34.67	13	4.74	0.714
**Level of trust regarding the management of COVID-related issues for PwMS** ***(mean, SE)***										
Treating neurologist	6.25	0.14	5.93	0.30	0.32	6.37	0.07	6.36	0.18	0.935
MS centers	5.60	0.18	4.87	0.39	**0.07**	5.55	0.10	5.54	0.32	0.954
MS-related scientific organizations	5.37	0.18	4.80	0.43	0.17	5.43	0.10	5.43	0.32	0.991
Patients' organizations	4.74	0.21	3.93	0.38	**0.08**	4.82	0.12	4.82	0.35	0.999
Other medical practitioners	4.76	0.22	5.00	0.35	0.57	4.65	0.16	4.38	0.41	0.516
Company's doctor *(if applicable)*	3.79	0.37	3.43	0.65	0.67	3.58	0.26	2.86	0.67	0.394
Employer (if applicable)	3.33	0.39	2.67	0.67	0.49	3.25	0.20	2.29	0.75	0.204
Media	2.86	0.20	3.00	0.46	0.76	2.93	0.12	3.75	0.31	**0.014**
Public hospitals	5.35	0.18	5.47	0.45	0.79	4.99	0.11	5.64	0.31	**0.038**
Primary health care facilities	4.49	0.21	4.80	0.37	0.50	4.14	0.13	4.61	0.35	0.181
Private clinical	4.05	0.20	2.80	0.34	**0.00**	4.02	0.13	3.89	0.31	0.704
Ministry of health	4.84	0.24	5.00	0.43	0.76	4.79	0.13	5.25	0.35	0.189
Health professional organization	4.67	0.22	5.07	0.41	0.40	4.72	0.12	4.96	0.35	0.450
Schools	3.40	0.19	2.60	0.38	**0.06**	3.09	0.11	3.04	0.30	0.864
Universities	3.95	0.21	3.20	0.39	0.11	3.32	0.12	3.43	0.35	0.730
Kindergartens	3.00	0.21	2.47	0.48	0.27	2.74	0.11	2.21	0.25	**0.071**
Public transportation	2.53	0.19	2.00	0.26	0.18	2.54	0.11	2.46	0.31	0.800

*COVID-19, coronavirus disease of 2019; SARS-CoV-2, Severe Acute Respiratory Syndrome Coronavirus-2; MS, Multiple Sclerosis; PwMS, People with Multiple Sclerosis; DMT, Disease-Modifying Treatment; SE, Standard Error of mean. Bold values indicate p < 0.1*.

#### COVID-19 and MS: MS Healthcare

**Figure 3:**
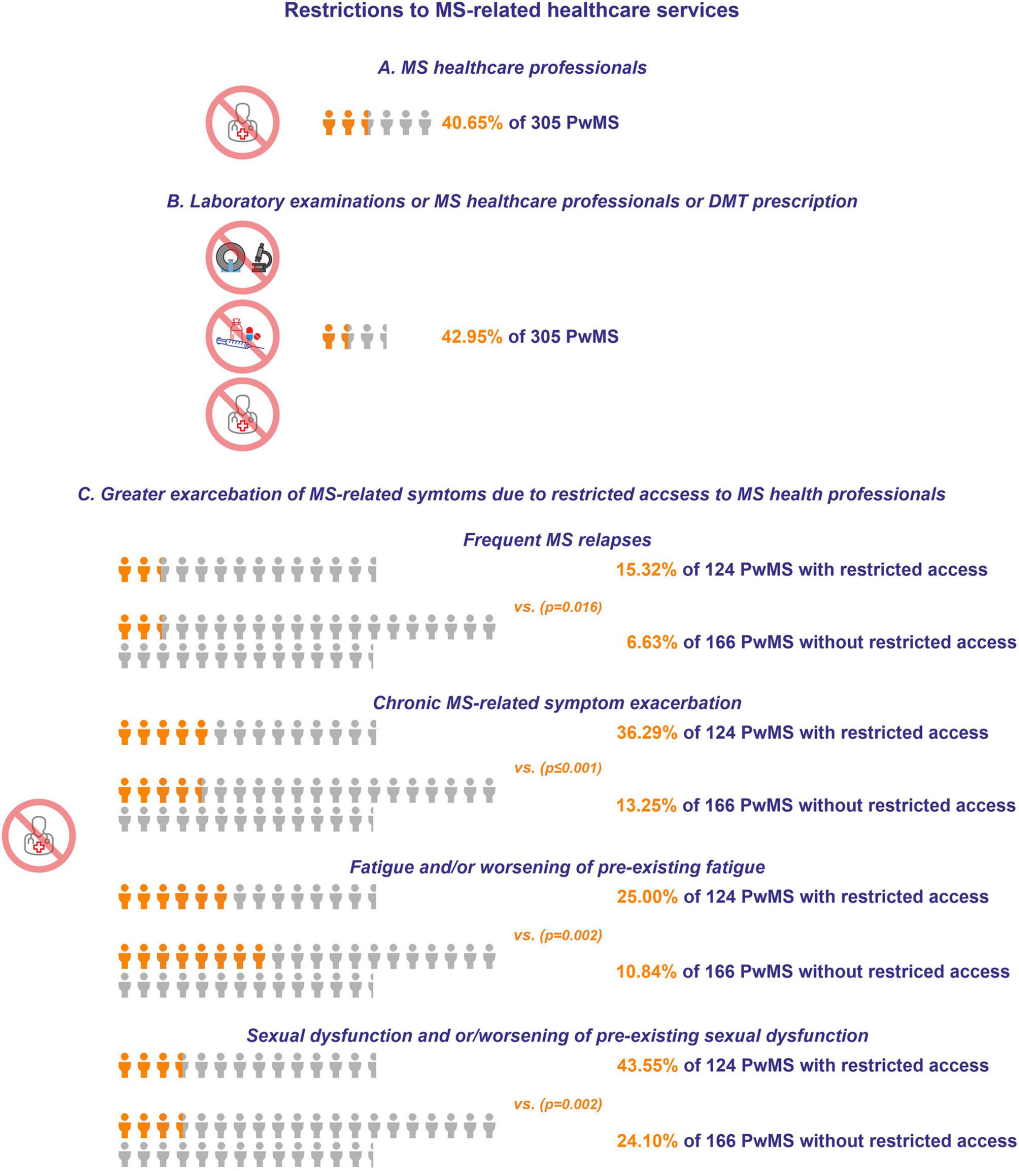
**(A)** Restrictions to MS healthcare professionals. **(B)** Restrictions to laboratory examinations or MS healthcare professionals or DMT prescription. The orange icon of human form depicts a significant portion of the respondents who experienced restrictions to MS-related healthcare services, while the light gray icon depicts the total number of respondents. **(C)** Exacerbation of MS-related symptoms with or without restricted access to MS health professionals. The orange icon of human form depicts the percentage of the respondents with exacerbation of MS-related symptoms, while the light gray icon depicts the total number of PwMS.

One hundred twenty-four (40.65%) PwMS out of 305 respondents for Part 3 of Questionnaire A, reported that they experienced restricted access to MS-related health professionals, due to the pandemic (Figure 3A). When taking into account additionally reported restricted access to DMT prescription and/or restricted access to MS-related laboratory examination, 131 (42.95%) PwMS reported restricted access to at least one category (Figure 3B). Data stratification with respect to gender and age is presented in Table 5 for 274 participants who participated in Part 1 and 3 of Questionnaire A. The PwMS who reported restricted access to MS-related health professionals due to the pandemic were on average of older age (46.18 ± 1.07 vs. 43.23 ± 0.93, *p* = 0.04), considered themselves more susceptible toward serious COVID-19 infection (5.6 ± 0.12 vs. 5.24 ± 0.13, *p* = 0.044), and they expressed more frequent thinking of the pandemic (3.57 ± 0.14 vs. 4.21 ± 0.13, *p* = 0.001), more overall SARS-CoV-2-related fear (3.44 ± 0.17 vs. 4.03 ± 0.15, *p* = 0.012), worry (2.84 ± 0.17 vs. 3.36 ± 0.14, *p* = 0.019), and stress (3.28 ± 0.17 vs. 3.81 ± 0.15, *p* = 0.023), compared with PwMS who did not report restriction to MS-related health professionals. The disease duration, type of MS, and PDDS score were not related to the reported access restriction. Moreover, the type of DMT by itself (first-line: glatiramer acetate, interferon-β, dimethyl fumarate, teriflunomide; second-line: natalizumab, fingolimod; third-line and/or highly-active treatments: ocrelizumab, alemtuzumab, cladribine) was not associated with restricted access to DMT (restricted vs. non-restricted: 22 vs. 122, 3 vs. 50 and 1 vs. 7 for each DMT category, respectively, *p* = 0.198). Participants under third-line and/or highly-active DMTs exhibited a tendency for increased fear related to the pandemic compared with the participants under first-line DMTs (4.9 ± 0.44 vs. 3.63 ± 0.14, *p* = 0.07). Moreover, the type of DMT category was also associated with a tendency for different levels of perceived sensitivity upon potential SARS-CoV-2 infection (5.22 ± 0.12 vs. 5.68 ± 0.16 vs. 5.82 ± 0.38 for each DMT category, respectively, *p* = 0.066). The PwMS who self-reported depression as comorbidity also reported reduced access to appointments with MS specialists compared with PwMS who did not self-report depression [access restriction yes/no (yes % of total): 7/8 (87.5%) vs. 73/183 (39.89%) respectively, *p* = 0.008]. Critically, subgroups of PwMS with restricted access to MS-related health professionals experienced several symptoms in greater exacerbation than PwMS who did not report such restrictions. Specifically, they experienced more frequent MS relapses during the 2 weeks prior to their participation in the study (19/124: 15.32% vs. 11/166: 6.63%, *p* = 0.016), chronic MS-related symptom exacerbation (45/124: 36.29% vs. 22/166: 13.25%, *p* < 0.001), fatigue and/or worsening of pre-existing fatigue (54/124: 43.55% vs. 40/166: 24.1%, *p* = 0.002), and sexual dysfunction and or/worsening of pre-existing sexual dysfunction (31/124: 25% vs. 18/166: 10.84%, *p* = 0.002) (Figure 3C). Overall, reduced access to DMTs did not seem to contribute to higher relapse frequency compared with non-restricted access to DMTs [relapse yes/no (yes % of total): 2/27 (6.89%) vs. 24/201 (10.66%) respectively, *p* = 0.4]. Also, reduced access to DMTs did not seem to contribute to a higher frequency of chronic symptom exacerbation compared with non-restricted access to DMTs [chronic symptom exacerbation yes/no (yes % of total): 9/23 (28.12%) vs. 51/178 (22.27%) respectively, *p* = 0.461]. The results were similar when the overall access to MS-related services was taken into account (data not shown). The PwMS who self-reported depression as comorbidity also reported sexual dysfunction and or/worsening of pre-existing sexual dysfunction during the 2 weeks prior to their participation in the study compared with the PwMS who did not self-report depression [sexual dysfunction unknown/yes/no (yes % of total): 3/7/5 (46.67%) vs. 18/38/199 (14.9%) respectively, *p* < 0.001].

**Table 5:** COVID-19 and multiple sclerosis care, lifestyle, and occupation in relation to multiple sclerosis, during the pandemic.

**Participants' gender**	**Male**					**Female**				
**Participants' age (years)**	**≤55**	**%**	**>55**	**%**	***p***	**≤55**	**%**	**>55**	**%**	***p***
*n* Participants (% of 274)	57	20.80	15	5.47		174	63.50	28	10.22	
**Experienced difficulties or delays due to COVID-19** ***(n, %)***										
MS appointment	19	6.93	7	2.55	0.472	68	24.82	13	4.74	0.695
Access to DMTs	10	3.65	1	0.36	0.367	20	7.30	2	0.73	0.780
Laboratory tests	12	4.38	2	0.73	0.626	33	12.04	5	1.82	0.635
**MS relapse** *(n, %)*	4	1.46	2	0.73	0.373	19	6.93	2	0.73	0.127
**MS-related symptom exacerbation** *(n, %)*	11	4.01	5	1.82	0.265	39	14.23	9	3.28	0.438
**Fatigue/worsening of pre-existing fatigue** *(n, %)*	16	5.84	3	1.09	0.814	58	21.17	10	3.65	0.710
**Sexual dysfunction** *(n, %)*	6	2.19	4	1.46	0.254	31	11.31	4	1.46	0.049
**Occupational status** ***(n, %)***										
Employer/self-employed	7	2.55	1	0.36	0.926	19	6.93	1	0.36	** <0.001**
Employee/salary	29	10.58	5	1.82		75	27.37	3	1.09	
Worker without salary (family business or agricultural)	0	0	0	0		4	1.46	0	0	
I do not work	5	1.82	1	0.36		29	10.58	3	1.09	
Retired	14	5.11	8	2.92		26	9.49	20	7.30	
Other (e.g., steady income, student/household)	2	0.73	0	0.00		20	7.30	0	0.00	
I don't know/I don't answer	0	0.00	0	0.00		1	0.36	1	0.36	
**Unemployment status** ***(if applicable) (n, %)***										
Not working due to the effects of MS, with a disability allowance	12	4.38	7	2.55	0.122	25	9.12	9	3.28	** <0.001**
Not working due to the effects of MS, without a disability allowance	0	0.00	1	0.36		13	4.74	4	1.46	
Not working temporarily but in search of work	2	0.73	0	0.00		14	5.11	0	0.00	
Other	3	1.09	0	0.00		5	1.82	1	0.36	
I don't know/I don't answer	3	1.09	0	0.00		5	1.82	3	1.09	
**Working status** ***(if applicable) (n, %)***
Full time, about 40 h a week	32	11.68	6	2.19	0.367	79	28.83	4	1.46	**0.002**
Part-time, about 20 h a week	3	1.09	0	0.00		7	2.55	0	0.00	
Occasional employment	0	0.00	0	0.00		6	2.19	0	0.00	
I do not answer	1	0.36	0	0.00		6	2.19	0	0.00	
**Working status affected (if applicable)** *(n, %)*	27	9.85	4	1.46	0.395	76	27.74	1	0.36	**0.001**
Closed owned business temporarily	2	0.73	0	0.00	0.98	11	4.01	0	0.00	0.739
Closed owned business permanently	1	0.36	0	0.00		0	0.00	0	0.00	
The employing company has temporarily closed its headquarters and I continue to work from home	6	2.19	1	0.36		17	6.20	0	0.00	
The employing company has temporarily closed its headquarters and I am not working	2	0.73	0	0.00		12	4.38	0	0.00	
The employing company did not close its headquarters but I continue to work from home	9	3.28	3	1.09		13	4.74	0	0.00	
Other	3	1.09	0	0.00		8	2.92	0	0.00	
**Feeling that they should have stayed home** *(n, %)*	12	4.38	1	0.36	0.224	15	5.47	0	0.00	**0.001**

*COVID-19, coronavirus disease of 2019; MS, Multiple Sclerosis; DMT, Disease-Modifying Treatment. Bold values indicate p < 0.1*.

#### COVID-19 and MS: Lifestyle and Occupation

Out of 305 respondents in Part 3 of Questionnaire A, the higher percentage of PwMS were employed (*n* = 130, 42.63%), whereas 74 (24.26%) were retired, 41 (13.44%) were unemployed, and 30 (6.23%) were self-employed. The remaining participants either did not provide an answer to this question or they provided descriptions that did not fall under these broad categories. Of all the non-working PwMS (*n* = 139), 59 (42.45%) reported unemployment due to MS-related disability receiving public financial support, whereas 19 (13.67%) reported unemployment due to MS-related disability without receiving public financial support. Of all the working PwMS (164 answers), 137 (83.54%) reported full-time (~40 h-weekly) employment, whereas 14 (8.54%) reported partial (~20 h-weekly) employment, and 6 (3.66%) reported temporary working status. Also, working PwMS reported that the pandemic affected their occupational status in 123 (75%) cases. Of those who described the means that the pandemic affected their occupation (123 answers), 58 (47.15%) reported working from home, whereas 28 (22.75%) reported that they either closed their own company due to the pandemic (*N* = 14) or were unemployed (during their participation in the study) due to the employing company being closed because of the pandemic (*N* = 14). Moreover, 32 (26.02%) out of 123 PwMS that were attending their work (during their participation in the study), reported their belief that they should have remained at home due to the pandemic. Interestingly, 43 (34.96%) PwMS who continued attending their work (during their participation in the study) reportedly did not know or refused to answer this question. The data stratification with respect to gender and age is presented in Table 5 for the 274 participants who participated in Part 1 and 3 of the Questionnaire.

### Vaccination Against COVID-19 (Questionnaire B)

#### Demographics

**Figure 4:**
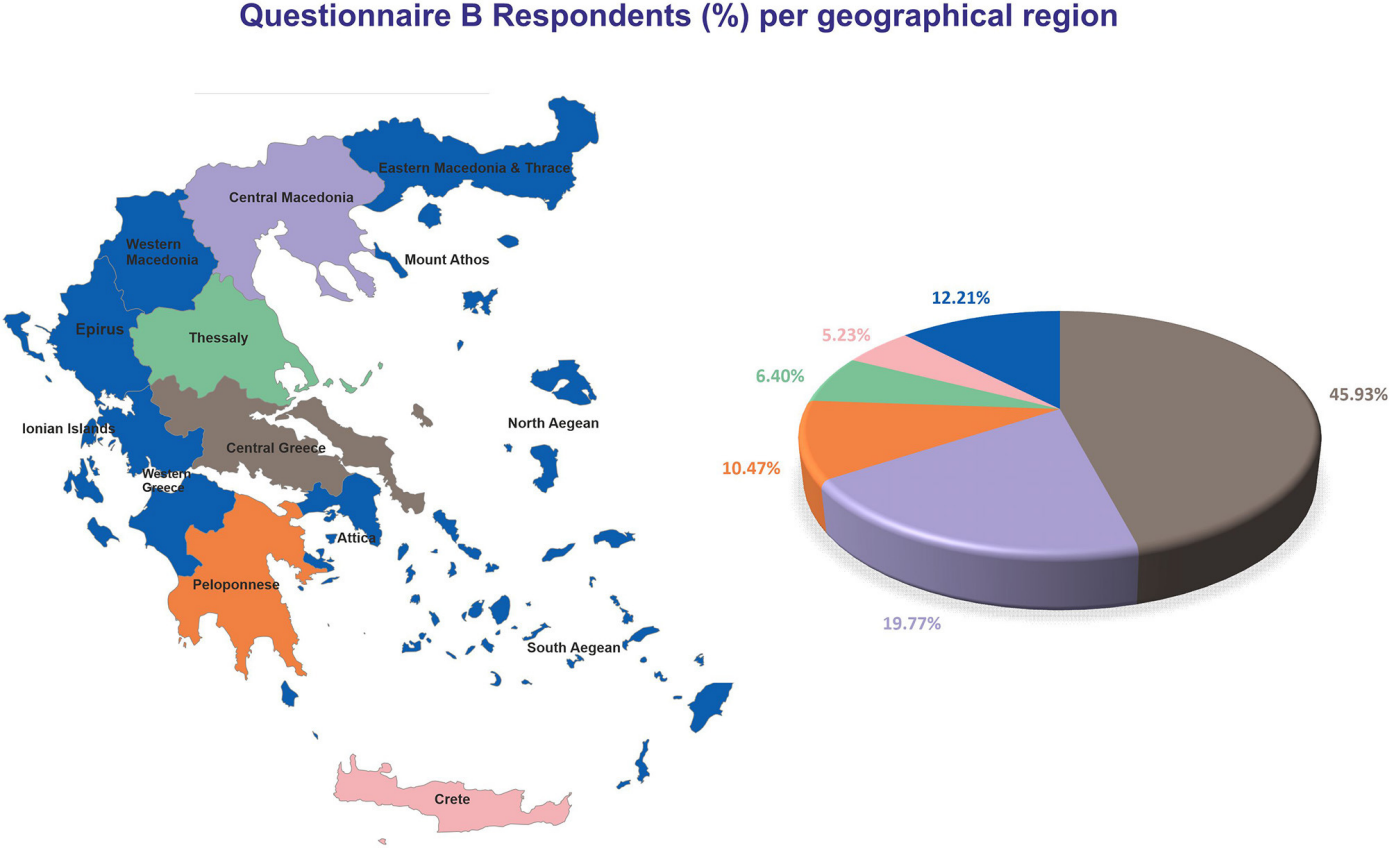
Geographical distribution of respondents for Questionnaire B.

Prior to the quality control, 183 answers were received for Questionnaire B. Following quality control, 176 responses from PwMS were considered for Questionnaire B (44 men; 132 women) with an average age of 47.64 ± 1.76 for men vs. 44.86 ± 0.97 for women (*p* = 0.157). One hundred seventy-two participants lived in Greece, 1 participant lived in Cyprus, and 3 participants lived abroad. The majority of the participants lived in Central Greece (*N* = 79), followed by Macedonia (*N* = 34), Peloponnese (*N* = 18), Crete (*N* = 11), and Thessaly (*N* = 9) (Figure 4).

#### MS-Related Medical History

The mean disease duration was 15.39 ± 1.65 years for men and 15.65 ± 0.88 for women (*p* = 0.886). The majority of participants reported a disease type of RRMS (*N* = 85), followed by SPMS (*N* = 31), and Primary Progressive MS (PPMS) (*N* = 24). One hundred forty-one PwMS reported receiving DMT at the time of participation, the most frequently administered DMT being fingolimod (*N* = 30), followed by dimethyl fumarate (*N* = 28), interferon-β (*N* = 23), glatiramer acetate (*N* = 19), natalizumab (*N* = 13), teriflunomide (*N* = 9), ocrelizumab (*N* = 5), alemtuzumab (*N* = 2), and cladribine (*N* = 2). Seventy-one PwMS reported PDDS score 0, whereas the remaining participants reported PDDS scores approximately equally distributed across the disability scores of 1–7.

#### SARS-CoV-2 Exposure and Vaccination Awareness

Five participants tested SARS-CoV-2 positive themselves (two PwMS with RRMS, two PwMS with SPMS, and one PwMS with an unknown form of the disease), whereas 76 participants reported having a close friend or relative that had been tested SARS-CoV-2 positive. There was no gender difference with respect to the self-reported degree of knowledge regarding vaccination against COVID-19 (4.86 ± 0.25 vs. 4.74 ± 0.14, respectively, *p* = 0.661). Compared with men, women self-reported higher degrees of knowledge with respect to vaccination against COVID-19 and MS (4.11 ± 0.16 vs. 3.41 ± 0.27, *p* = 0.03), and they reported a higher degree of trust in their treating neurologist (5.95 ± 0.11 vs. 3.41 ± 0.27, *p* = 0.05). Men and women did not differ with respect to their level of trust toward other sources of information regarding vaccination against COVID-19 and MS, such as scientific organizations, MS centers, MS sites, PwMS organizations, consultation with other MS-related healthcare providers, other PwMS, and/or social media (data not shown).

#### COVID 19 and MS: Vaccination Adherence

**Figure 5:**
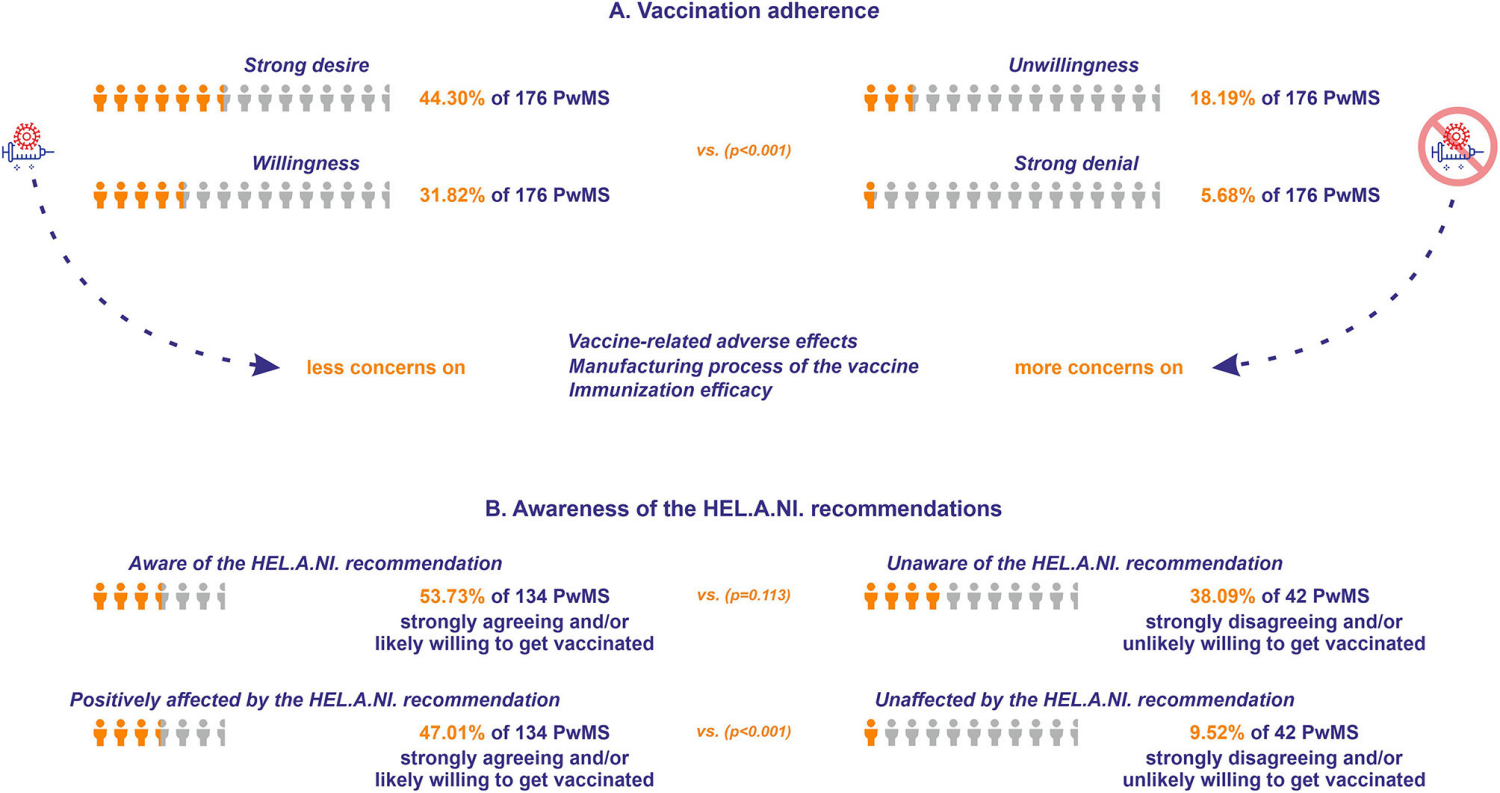
**(A)** Vaccination adherence for the respondents of Questionnaire B. The orange icon of human form depicts the percentage of the respondents strongly agreeing or likely willing to get vaccinated (vs. those strongly disagreeing or unlikely willing), while the light gray icon depicts the total number of PwMS who responded to the questionnaire. **(B)** Awareness of vaccination recommendations for the respondents of Questionnaire B. The orange icon of human form depicts the percentage of the respondents who were aware of the Hellenic Academy of Neuroimmunology (HEL.A.NI.) recommendation (vs. those unaware), and the respondents who were positively affected by the HEL.A.NI. recommendation (vs. those who were unaffected). The light gray icon depicts the sum of the respondents strongly agreeing and/or likely willing, and the sum of the respondents strongly disagreeing and/or unlikely willing to get vaccinated.

The majority of the participants reported a strong desire to be vaccinated against COVID-19 (*N* = 78), followed by participants who reported that they were likely willing to be vaccinated (*N* = 56). Thirty-two participants likely did not want to be vaccinated and 10 strongly disagreed with vaccination against COVID-19. The relative distribution of answers did not differ between men and women (*p* = 0.31). When the participants were divided between those that were strongly agreeing and/or likely willing to undergo vaccination against COVID-19 and those that were strongly disagreeing and/or unlikely willing to undergo vaccination, the former reported significantly less concern with respect to the vaccine-related adverse events compared with the latter (3.69 ± 0.17 vs. 2.21 ± 0.28, respectively, *p* < 0.001), with respect to the manufacturing process of the vaccine (4.47 ± 0.17 vs. 2.69 ± 0.31, respectively, *p* < 0.001), as well as with respect to the immunization efficacy (4.31 ± 0.17 vs. 2.57 ± 0.24, respectively, *p* < 0.001) (higher mean scores denote lower concern) (Figure 5A).

#### COVID 19 and MS: Vaccination Recommendations and Immunization

Seventy-two participants willing (strongly agreeing and/or likely willing) to get vaccinated were aware of the recent HEL.A.NI. recommendation (33) concerning MS and DMTs and vaccination against COVID-19, vs. 16 participants non-willing (strongly disagreeing and/or unlikely willing) to undergo vaccination (*p* = 0.113). Sixty-three participants willing to be vaccinated were positively affected in their decision by the recent HEL.A.NI. recommendation, vs. four participants non-willing to be vaccinated (*p* < 0.001; Figure 5B). Sixteen participants were vaccinated against COVID-19 at the time of participation (15 with the Pfizer/Moderna vaccine and one with the AstraZeneca vaccine). Eight participants received two doses and the rest received one dose of the vaccine. Two participants underwent antibody anti-SARS-CoV-2 titer evaluation, one reporting satisfying immunization (patient on fingolimod), whereas the other reported reduced efficacy of the immunization (patient on natalizumab).

## Discussion

People with MS are in constant need and thus often seek guidance regarding the impact of their disease and medication on their risk of COVID-19. The present study investigated the effects of the COVID-19 pandemic in several aspects of the quality of life, the behaviors regarding COVID-19 and MS, as well as the healthcare and the working conditions of PwMS in Greece. This is a nationwide study conducted by members of the HEL.A.NI. scientific association in cooperation with AUTH and the HFoPwMS. The results presented here contribute to the overall assessment of the impact of the COVID-19 pandemic in a representative population of PwMS, as indicated by the geographical distribution of MS cases in Greece, recently published by our group (26). The first COVID-19 questionnaire of HEL.A.NI. is part of the MSDA catalog, implicating that the metadata and descriptive information about the questionnaire is cataloged by the MSDA, which contributes to improving the findability and accessibility of real-world data and thereby facilitating collaborative research. Moreover, the vaccination questionnaire of HEL.A.NI. has been used in the COVID-19 vaccine mapping exercise in the frame of the global data sharing initiative (GDSI) of the MSDA (23), which in turn contributed to the creation of the recommended minimal dataset on COVID-19 vaccination, thus contributing to aligning data collection, an important step to facilitate collaborative research.

In our study, increased age, longer disease duration, and higher MS-related disability were self-identified as potential risk factors toward SARS-CoV-2 infection, and the perceived severity of COVID-19 upon potential infection. Critically, subgroups of PwMS, which reportedly experienced restricted access to MS-related health services due to the pandemic, experienced several symptoms in greater exacerbation than the PwMS who did not report such restrictions. Interestingly, self-reported relapses and/or symptom exacerbation were not associated with restricted access to DMTs. This finding may be, at least in part, attributed to the majority of the answers received at the initial stage of the pandemic when the result of the potentially restricted access to DMTs would require a more extended period to lead to an increase in relapses and/or disease deterioration. Moreover, chronic MS symptom exacerbation does not imply true relapse activity and may, therefore, be attributed to factors distinct from DMT access, such as a reduction in physical activity, as expected in circumstances of social isolation and quarantine. However, this parameter was not evaluated in the frame of the present study. Notably, the attitudes toward vaccines and intention to vaccinate against COVID-19 were positive.

The majority of the participants were highly educated, which may account for their possible above-average knowledge regarding the COVID-19 pandemic, lived in urban centers, and exhibited relapsing-remitting forms of MS. At the time of the study which spanned across all three waves, the majority was already aware of the pandemic and was informed about the spreading prevention measures. At the time of the survey, they continued being on DMT. A minority of them reported comorbidities, mainly autoimmune thyroid disease and depression. Depression as self-reported comorbidity exhibited a partial effect with respect to specific outcomes, such as the frequency of restricted access to appointments with MS specialists and behaviors, such as sexual dysfunction. Our findings are consistent with existing literature underlining a higher risk of anxiety and depression for PwMS relative to the general population and that these feelings are related to poor well-being during the pandemic (34). It should be stated, however, that in the frame of the present study, depression was not assessed by an online self-reported assessment tool, therefore a safe conclusion on the basis of discriminating the effect of current self-reported depressive feeling vs. a history of clinical diagnosis of depression on the reported outcomes is not possible. Only a few of the participants reported personally knowing someone exposed to SARS-CoV-2. However, this fact was not related to their anxiety regarding the pandemic, compared with those who did not know someone exposed to the virus. Older age, longer disease duration, and higher MS-related disability were associated with increased consciousness regarding the perceived sensitivity toward SARS-CoV-2 infection, as well as the perceived severity of COVID-19 upon potential infection. Moreover, these PwMS expressed increased feelings of anxiety and overall insecurity regarding their ability toward self-protection and infection prevention. These results are in line with recent studies highlighting that PwMS of advanced age and with long disease duration are highly likely to experience increased levels of anxiety due to the pandemic (13, 14).

Younger PwMS (<50 years old) favored social media as the source of information regarding COVID-19, an observation in accordance with the general preferences of this age category regarding the source of information in general. However, with respect to the source of COVID-19 and MS-related information mostly trusted, the treating neurologist was considered as the most reliable source of information for all age groups of PwMS, as indicated previously (35).

A significant proportion of PwMS experienced restricted access to MS-related health professionals, DMT prescription, and/or to MS-related laboratory examination, due to the pandemic. The majority of them were of age older than 50 years. As a note of caution in the care of PwMS, this subgroup expressed more frequent feelings of anxiety, fear, insecurity, and worry about the pandemic compared with the PwMS who did not report similarly restricted access to MS-related healthcare services. These findings confirmed the results of two recent online cross-sectional surveys (12, 32) and highlighted that those difficulties in receiving MS-related healthcare services, psychological assistance, and rehabilitation therapy are related to negative psychological outcomes for PwMS, as it is reported for people with other chronic conditions. Interestingly, however, people with chronic diseases have been reported to exhibit a high likelihood of medical appointment cancellation during the pandemic and this behavior correlates with levels of COVID-19-related anxiety (36).

With respect to the occupational status of PwMS, the majority of the non-working PwMS reported unemployment due to MS-related disability. A significant percentage (75%) of employed PwMS reported that the pandemic affected their occupational status. The main reasons for the alterations in occupational status were reportedly, either working from home or that the employing company has closed down. People with MS are reportedly prone to joblessness and stressful work environments, as is expected during the COVID-19 pandemic, which may be of disadvantage for them (32). Interestingly, of those who continued attending their work, a significant proportion either reported that they should have remained at home or refused to answer, thus, possibly denoting increased anxiety due to the pandemic. Of note, the working conditions prior to the pandemic were not evaluated in the frame of the present study, a condition that may pose a significant effect on the studied outcomes. One study previously addressed the employment status for 200 PwMS in Greece, reporting a low proportion as 32% of PwMS are being fully or partially employed (37). The extent of the administered questionnaire did not allow for a detailed evaluation of the working conditions in the context of MS for the participants. Recent work by our group has shown that age, level of education, and partial-time occupation status may be linked with work barriers for PwMS (31).

The majority of the participants reported either strong willingness toward or that they were likely willing to be vaccinated against COVID-19. Adverse events following vaccination against COVID-19 were the most highly ranked source of concern among those who strongly and/or likely refused vaccination, followed by concerns regarding immunization efficacy, as well as the manufacturing process of the vaccine. Being aware of the HEL.A.NI. recommendations regarding COVID-19 vaccination for PwMS reportedly affected the willingness of the participants to receive the vaccine. In line with existing literature (38, 39), this observation further underlines the necessity of scientific organizations to take joint action with patient organizations to increase awareness with respect to accurate and up-to-date guidance on health-related issues during the pandemic.

Our study is subjected to limitations, such as the inherent restriction to ICT illiterate participants unless a caregiver was acting on their behalf to complete the survey according to the instructions of the participant. In close relation, the length of Questionnaire A has discouraged PwMS who are prone to cognitive fatigue to complete it in total, despite being divided into three parts. It is therefore likely that the overall long completion time for Questionnaire A contributed to the missed submissions, especially for Parts 2 and 3. Moreover, although the questionnaire was accessible since April 2020, patients' main pool of answers came from one time period, April–May 2020, whereas significantly fewer answers were received from June until the end of 2020. Admittedly, these two periods are different in terms of knowledge of the disease: in April 2020 the pandemic was at an initial stage, therefore the illness in itself was not well-characterized. In the following months, more information regarding the transmission and the disease course was available. However, comparative analysis across the time points was not conducted in the overall setting of the present study, as it was unlikely to yield safe conclusions with respect to the evolution of participants' perceptions and behaviors concerning COVID-19. Few results presented in this setting did not show a significant difference in several measurements of self-reported frequent thinking of the pandemic and the related worry and anxiety but these results pose limitations taking into account the uneven number of answers between time-points. In addition, the recruitment method limited the pool of participants, mainly to the members of the HFoPwMS, especially those more active and responsive to online communication tools and initiatives. However, this is an inherent limitation of the implementation of ICT for research and data collection, thus underlining the need for increased public awareness and education regarding its use and the related scientific value of ICT-derived data.

In conclusion, the present study provides a thorough estimation of the perceptions and behaviors of PwMS, as well as their occupational and health-related alterations throughout the pandemic and in anticipation of immunization against COVID-19 at a national level. The constant effort of the scientific community to retain the accessibility of MS-related health services, as well as to establish open routes of communication with PwMS are of utmost significance in the struggle against the pandemic. Online ICT tools may prove especially useful in polling the perceptions and behaviors of PwMS and in delivering evidence-based scientific guidance, to facilitate timely decision making, increased awareness, and the overall success in the implementation of prevention measures and health policies during the pandemic. In the absence of a national MS registry in Greece, which is currently under development, the present initiative serves to facilitate active cooperation between stakeholders, namely, MS specialists, PwMS, as well as the respective scientific and patient organizations. As ours and several other worldwide initiatives continue to collect more data and contribute to MS registries, we expect soon to have a comprehensive outlook to further guide the management of PwMS.

## Data Availability Statement

The original contributions presented in the study are included in the article/Supplementary Material, further inquiries can be directed to the corresponding author.

## Ethics Statement

The studies involving human participants were reviewed and approved by the Bioethics Committee of the School of Medicine of the Aristotle University of Thessaloniki (Approval Nr. 6322/29-7-2020). The patients/participants provided their written informed consent to participate in this study.

## Author Contributions

MB and NG: conception and design of the study, acquisition of data, analysis and interpretation, critical revision of the manuscript for important intellectual content, and final approval of the version to be submitted. CS, CB, EG, A-SS, and IN: acquisition of data, analysis and interpretation, critical revision of the manuscript for important intellectual content, and final approval of the version to be submitted. AV, LG, S-HP, LP, GP, and PB: critical revision of the manuscript for important intellectual content, and final approval of the version to be submitted. All authors contributed to the article and approved the submitted version.

## Conflict of Interest

MB received travel support from Biogen Idec, Novartis, TEVA, Bayer, Merck, Genesis Pharma, Sanofi; Lecture fees from TEVA, Merck, Novartis, Genesis Pharma, Sanofi, Roche; Research grants from the Hellenic Foundation for Research and Innovation (H.F.R.I.), the Ministry of Education's Education and Lifelong Learning Program, the Hellenic Neurological Society, Biogen Idec, Novartis, TEVA, Genesis Pharma; Consultancy fees from Merck. CB received travel support and/or research grants and/or lecture fees from Novartis. Bayer, Merck, Genesis, Sanofi, Teva, Roche, Biogen, and Mylan. IN received conference fees and/or travel support, and/or speaker honoraria, and/or honoraria for participation in advisory boards and/or grants/research support: Bayer, Specifar- Teva, Novartis, Sanofi-Genzyme, Roche, Mylan, Merck, Genesis Pharma, Hellenic Foundation for Research and Innovation (H.F.R.I.), and the General Secretariat for Research and Innovation (G.S.R.I). LG was funded by the Flemish Government under the Onderzoeksprogramma Artificiële Intelligentie Vlaanderen. NG received Travel support and/or research grants and/or lecture fees and/or advisory services: Novartis, Bayer, Merck, Genesis, Sanofi, Specifar, Roche, Biogen, TEVA, Mylan. The remaining authors declare that the research was conducted in the absence of any commercial or financial relationships that could be construed as a potential conflict of interest.

## Publisher's Note

All claims expressed in this article are solely those of the authors and do not necessarily represent those of their affiliated organizations, or those of the publisher, the editors and the reviewers. Any product that may be evaluated in this article, or claim that may be made by its manufacturer, is not guaranteed or endorsed by the publisher.
