# Supplementary material for: A National Representative, Cross-Sectional Study by the Hellenic Academy of NeuroImmunology (HEL.A.NI.) on COVID-19 and Multiple Sclerosis: Overall Impact and Willingness Toward Vaccination
Source: Front Neurol. 2021 Nov 25;12:757038. doi: 10.3389/fneur.2021.757038 (PMC8656423; doi:10.3389/fneur.2021.757038)
Supplement: Supplementary file 1 [file Data_Sheet_1.docx]

**Annex 1**

**Introduction page of the online Questionnaire A**

Dear Participant,
Thank you for taking part in this study.
This study is an initiative of the Hellenic Academy of Neuroimmunology ([HEL.A.NI](https://www.google.com/url?q=http://HEL.A.NI&sa=D&source=editors&ust=1626695367945000&usg=AFQjCNFC1cInlHhaf9OdFmUssxg6RQTjiw).) in collaboration with the Laboratory of Medical Physics of the Aristotle University of Thessaloniki (AUTH) in the frame of the international effort currently underway in order to capture the effects of the novel coronavirus (COVID-19) pandemic in people with Multiple Sclerosis (PwMS) and their caregivers.
This questionnaire is an adaptation from:
WHO Regional Office For Europe. (2020). COVID-19 Snapshot MOnitoring (COSMO Standard): Monitoring knowledge, risk perceptions, preventive behavior, and public trust in the current coronavirus outbreak - WHO standard protocol. PsychArchives. [https://doi.org/10.23668/PSYCHARCHIVES.2782](https://www.google.com/url?q=https://doi.org/10.23668/PSYCHARCHIVES.2782&sa=D&source=editors&ust=1626695367945000&usg=AFQjCNH2FSN3G1rkguI4AXyHL_bgEomy8g)
This item is licensed under a Creative Commons License.
[https://creativecommons.org/licenses/by-sa/4.0/](https://www.google.com/url?q=https://creativecommons.org/licenses/by-sa/4.0/&sa=D&source=editors&ust=1626695367945000&usg=AFQjCNHYvAgC57bQrqsjcX6Ld90t_gV0Mg)

In order to register to the system as you will see below we require the submission of your email address. The reason for this information request is to ensure the reliability of the answers to the questionnaire and that the data entered into the system are not random. In essence, however, none of your personal data, general and sensitive, will be available to HELANI, as it will be anonymized to a following step (see next page), thus eventually making your participation anonymous.
The total time to complete the questionnaire is estimated to be about 60 minutes. For your convenience, we have divided the questionnaire into three parts:
Part 1: questions on general information and Multiple Sclerosis regarding you or the person with Multiple Sclerosis that you take care of (about 15 minutes)
Part 2: questions about your beliefs and your daily behaviors regarding the novel coronavirus COVID-19 (about 25 minutes).
Part 3: Questions concerning your beliefs regarding the novel coronavirus COVID-19 in relation to Multiple Sclerosis and the effect of the COVID-19 pandemic on the management of Multiple Sclerosis for you or for the person with Multiple Sclerosis that you take care of (20 minutes).
Each Part is submitted separately. Please do not begin with a Part unless you have enough time to complete it and also make sure that you press "Submit" at the end of each Part, as the system does not allow temporary storage and submission at a later time. Close other programs (eg chat or email) in order to avoid distraction.
It is necessary to complete all three Parties in order for us to take your answers into account and to draw conclusions.
The study is conducted to improve actions taken at a national level in response to the novel coronavirus pandemic in relation to MS, while at the same time it is expected to contribute to the World community's efforts to tackle the current and potential future pandemic more effectively.
Your answers will be used exclusively for scientific purposes and will help to improve the response of the scientific community against the novel coronavirus outbreak in relation to MS.
If you are having trouble handling your PC, please contact a caregiver / legal representative to fill out the questionnaire. In this case the filling should be done in real time, during which the caregiver will complete the answers you indicate. If the study is conducted through a caregiver / legal representative please state this in the appropriate field.
This is a non-commercial study for which no funding was received.


Further information:
1. The anonymized data will be stored permanently and published for possible later use by other scientists. Conclusions about your or other persons are not possible.
2. Your data will be treated in accordance to the regulations of the European Data Protection Regulation (GDPR EU).
3. Your participation in the study is voluntary and can be terminated at any time and without providing specific reason.
4. Your participation in the research does not expose you to any significant risk.

Why we collect and use your data
The study about the novel coronavirus (COVID-19) and is designed for People with Multiple Sclerosis (PwMS) and their caregivers. The anonymized data will be permanently stored and will be submitted to a Repository for future potential use by other researchers in the future.

There are no right or wrong answers, we are interested in recording your experience.
Thank you

How you can contact us
Hellenic Academy of Neuroimmunology
Politechniou 23, PC 54625,
Thessaloniki, Greece
Τelephone:+30 2313 050 426
[info@helani.gr](mailto:info@helani.gr)


If you would like more information on the processing of your personal data, click on the following link [https://drive.google.com/open?id=1X3GYf5Yh7oJVvVQwmzWfQloK7tbGjlxx](https://www.google.com/url?q=https://drive.google.com/open?id%3D1X3GYf5Yh7oJVvVQwmzWfQloK7tbGjlxx&sa=D&source=editors&ust=1626695367945000&usg=AFQjCNFZbz3q1HZJcMuCVKWw9_pNgOC7Xg)

* Απαιτείται

Διεύθυνση ηλεκτρονικού ταχυδρομείου *

Η διεύθυνσή σας ηλεκτρονικού ταχυδρομείου

Informed Consent *

I declare that I have been informed with respect to the information note regarding the collection, processing and storing of my personal data.

I agree with the processing of my personal data for the purposes of reliability of the research.

I am aware that I may revoke my consent at any time until the deletion of my registration data and the anonymization of my answers to the inquiry.

**Privacy notice and Consent Declaration**

**Contents**

Introduction............................................................................................................. 2

Who is responsible for the protection of your personal data (Data Controller); .............................................................................................................. 2

Type of Data processed:.................................................................................... 2

What is the purpose and the lawful basis for processing your personal data?......................................................................................................................... 3

Who has access to your data? What measures of protection has HELANI undertaken?............................................................................................. 4

Period of time during which personal data is processed.......................... 4

What are your data protection rights?........................................................... 4

Right to be informed: .......................................................................................... 5

Right to access: .................................................................................................... 5

Right to rectification:............................................................................................ 5

Right to erasure:..................................................................................................... 5

Right to restrict processing: ................................................................................ 5

Right to object to processing: ........................................................................... 5

Right to portability:................................................................................................. 6

Right to revoke consent:.......................................................................................6 Exercising your rights: ........................................................................................... 6

Right to file a complaint with the DPA............................................................. 6

Introduction

We would like to inform you that the protection of your personal data is of the utmost importance for the **HELLENIC ACADEMY OF NEUROIMMUNOLOGY (HELANI)**.

This notice refers exclusively to **the research carried out in order to improve the measures taken for the protection of patients with MS from the new COVID-19.**

This study is conducted by the Hellenic Academy of Neuroimmunology (HELANI). This study concerns the new coronavirus (COVID-19) and is addressed to patients with Multiple Sclerosis (MS) and carers of patients with MS. Your answers to the study’s questions are kept separately from your identification data, so that they can be used by the scientific team without incurring any risk to the personal data of participants.

Through the present document, you are asked, once you have read the detailed notice that follows, to provide your consent for the processing of your personal data which are necessary to conduct this research.

Who is responsible for the protection of your personal data (Data Controller)?

The non-profit civil partnership HELLENIC ACADEMY OF NEUROIMMUNOLOGY (seated in Thessaloniki, Politechneioy str., nr. 23, email: info@helani.gr tel.: +30 2313 050 426, website: helani.gr)**, hereinafter HELANI,** legally represented, wishes to inform you that it collects information which constitute personal data of the participants, in compliance with national legislation and European Regulation GDPR 679/2016, (hereinafter “Regulation” »), for the purposes of its study to improve the measures taken for the protection of patients with MS from the new COVID-19.

HELANI as Data Controller, **collects, processes and stores** the following personal data **exclusively** for the following purposes:

Type of Data processed:

**Α) Ordinary Data, especially identification and contact particulars such  as:**

- phone number, postal and e-mail address,

- age

- sex

- level of education

- geographical region of place of residence

- profession

- family status

**Β)Special Category Data (Sensitive data)**

Health data (e.g. current health situation, medication etc.)

What is the purpose and the lawful basis for processing your personal data?

In order to participate in this study you will have to provide information in  two stages:

1) during your registration. At this point you will have to provide your identification data (e-mail address). The exclusive purpose of collecting this data is to check the study’s reliability, and to confirm that people that are not patients with MS or carers of patients with MS do not participate. This data is collected only upon your consent (article 6 par.  1a GDPR). The doctors participating in the main part of the study do not have access to this data.

You have the right to freely recall your consent at any moment and up to their deletion, without prejudice to the lawfulness of the processing which was based on your consent prior to you recalling it.

2)when answering the questionnaire: the data that you fill in when answering the study’s questions.

This data is not linked to your identification data that you provided during your registration and are kept separately.

The purpose of the collection of the study’s health data is:

- the establishment of statistical data per geographical region for the extraction of conclusions regarding the level of preparedness, the practical problems vis-à-vis access to treatment and the level of knowledge to combat coronavirus COVID-19.

The legal basis of this processing is the overriding legitimate interest of HELANI to conduct scientific and statistical studies in order to formulate guidelines and proposals for the protection of patients with MS and is allowed because it is necessary to conduct research for statistical purposes (articles 6 par. 1f & 9 par.2j GDPR, article 30 of L.4624/2019).

- the use of the study’s results for the formulation of scientific proposals in order to improve the protection from coronavirus COVID-19.

The legal basis of this processing is the overriding legitimate interest of HELANI to conduct scientific and statistical studies in order to formulate guidelines and proposals for the protection of patients with MS and is allowed because it is necessary to conduct research for statistical purposes (articles 6 par. 1f & 9 par.2j GDPR, article 30 of L.4624/2019).

You have the right to object at any time to the processing of your personal data where it is necessary for the purposes of legitimate interests of HELANI, such as the processing of your answers to the questions of the study. This right may be exercised until the anonymization of your answers and will be complied with if said right  supersedes the legitimate interests pursued by this study.

Who has access to your data? What measures of protection has HELANI undertaken?

In order to collect all the necessary data for this study, HELANI is collaborating with the Laboratory of Medical Physics of AUTH. The laboratory uses its informatics department to collect and safely store the identification data provided during your registration, without granting access to anyone else (including HELANI). The laboratory uses secure

data hosting services.

Regarding the questionnaires you have submitted, they do not contain any identification data (e-mail), and only those are directed to HELANI in the form of an excel file that does not include any data, or digital footprint information, that can directly identify you. These files are used

by HELANI anonymously and for the purposes of the study. All the conclusions and proposals of the study that will be published by the scientific team of HELANI will only include anonymous data. The anonymous collective results of the study may be useful for future scientific research as well.

Your data are not transferred to recipients outside of the EEA, that do not guarantee an adequate level of personal data protection. HELANI has taken every suitable measure for the protection of your personal data such as pseudonymization, data encryption or restricted access to said data.

The answers to the study will be anonymized upon the conclusion of the study’s purposes. Anonymisation is a technique applied to personal data with the aim of irreversibly preventing identification of the data subject in such a way that the subject’s identity can no longer be identified directly or indirectly, even when all the means that can be reasonably used for identification either by the controller or by any third party are used.

Period of time during which personal data is processed

Regarding the data provided during your registration (e-mail): they are stored for as long as it is necessary for the needs of the study and until the analysis and extraction of results. They are then safely deleted and cannot be recovered.

Regarding the data submitted during the survey: they are not linked to your registration data. Following the deletion of your registration data and the completion of the study’s purpose, the data is then anonymized making it impossible to identify the participant and are no longer considered personal data. The anonymized data is kept for 10 years.

What are your data protection rights?

Each person whose data is processed by us enjoys a number of rights which are detailed below. Especially in regards to the rights to be informed, to rectification, to object and to erasure, they are subjected to the limitations set by law, should their exercise possibly prevent or make it much more difficult to achieve the purpose of the study and if these

limitations are considered necessary for their fulfillment. As such, article 15 of the GDPR and the subject’s right to access is not applicable when the personal data are necessary for scientific purposes and the provision of information requires disproportionate effort on our part, as for example in the case where we have erased your identification data. In order to

make compliance to your rights easier until the anonymization of the questionnaire, you are requested to declare an e-mail which you must retain in order to exercise your rights in the future.

Right to be informed:

We are obliged to inform you of the ways in which your data is being used, e.g. which data is being processed, to which end, for how long the data is kept, in a concise, transparent, intelligible and easily accessible form, using clear and plain language,

Right to access:

You have the right to access the data and receive additional information about how they are processed.

Right to rectification:

You have the right to study, correct, update or modify your data.

Right to erasure:

You have the right to request your personal data to be erased. However, the right to have data erased does not exist if the erasure prevents the purpose of processing from being fulfilled for scientific research purposes or makes it much more difficult.

Right to restrict processing:

You have the right to request a limitation on the processing of your  personal data in the following cases: (a) when you contest the accuracy  of your personal data and until it has been verified, (b) when you object  to the erasure of personal data and you request that their use is limited  as opposed to erased c) when personal data is not needed for  processing purposes, it is, however, indispensable for the foundation,  exercise, support of legal claims, and (d) when you object to the  processing and until it is verified that there are legitimate grounds that  concern us and supersede the reasons for which you object to the  processing.

Right to object to processing:

You have the right to object at any time to the processing of your personal data where it is necessary for the purposes of legitimate

interests of HELANI, such as the processing of your answers to the questions of the study.

This right may be exercised until the anonymization of your answers and will be complied with if said right supersedes the legitimate interests pursued by this study.

Right to portability:

You have the right to receive your personal data free of charge in a format that allows you to access, use, and edit it with commonly used editing methods. You also have the right to ask us, if technically feasible, to pass the data directly to another controller. This right exists for the data you have provided to us and is processed by automated means based on your consent or performance of a relevant contract.

Right to revoke consent:

We would like to inform you, that the processing of your registration data (e-mail) is based on your consent and as such you have the right to recall it freely, without prejudice to the lawfulness of the processing which was based on your consent prior to you recalling it. This may take

place up to the deletion of this data by HELANI.

Exercising your rights:

Once your initial registration data has been deleted, it will not be possible to identify you and comply with your requests regarding said data. In regard to your answers in the study and until their anonymization, you will have to retain the e-mail you declared with the system, in order to verify your identity and comply with your requests, in line with the restriction set by law and as mentioned above.

In order to exercise any of the above rights you may submit your request to the following contact details:  
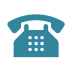


email: info@helani.gr

tel.: +30 2313 050 426

Right to file a complaint with the DPA

You have the right to file a complaint with the Data Protection Authority (www.dpa.gr): Call Centre: +302106475600, Fax: +30210 6475628, Email:  complaints complaints@dpa.gr.

**Consent Declaration:**

**I declare that I have read the information notice regarding the collection, processing and storage of my personal data. I consent to the processing of my personal data for the purpose of the study’s reliability.**

**I am aware that I may recall my consent at any time up to the deletion of my registration data and the anonymisation of my answers to the study’s questionnaire.**
